# Investigating the association between glycaemic traits and colorectal cancer in the Japanese population using Mendelian randomisation

**DOI:** 10.1038/s41598-023-33966-7

**Published:** 2023-04-29

**Authors:** Akiko Hanyuda, Atsushi Goto, Ryoko Katagiri, Yuriko N. Koyanagi, Masahiro Nakatochi, Yoichi Sutoh, Shiori Nakano, Isao Oze, Hidemi Ito, Taiki Yamaji, Norie Sawada, Masao Iwagami, Aya Kadota, Teruhide Koyama, Sakurako Katsuura-Kamano, Hiroaki Ikezaki, Keitaro Tanaka, Toshiro Takezaki, Issei Imoto, Midori Suzuki, Yukihide Momozawa, Kenji Takeuchi, Akira Narita, Atsushi Hozawa, Kengo Kinoshita, Atsushi Shimizu, Kozo Tanno, Keitaro Matsuo, Shoichiro Tsugane, Kenji Wakai, Makoto Sasaki, Masayuki Yamamoto, Motoki Iwasaki

**Affiliations:** 1 Division of Epidemiology, National Cancer Center Institute for Cancer Control, 5-1-1 Tsukiji, Chuo-Ku, Tokyo, 104-0045 Japan; 2 Department of Ophthalmology, Keio University School of Medicine, Tokyo, Japan; 3 Department of Health Data Science, Graduate School of Data Science, Yokohama City University, 22-2 Seto, Kanazawa-Ku, Yokohama, 236-0027 Japan; 4 Division of Cancer Information and Control, Aichi Cancer Center, Nagoya, Aichi Japan; 5 Public Health Informatics Unit, Department of Integrated Health Sciences, Nagoya University Graduate School of Medicine, Nagoya, Aichi Japan; 6 Division of Biomedical Information Analysis, Iwate Tohoku Medical Megabank, Morioka, Iwate Japan; 7 Division of Cancer Epidemiology and Prevention, Aichi Cancer Center, Nagoya, Aichi Japan; 8 Division of Descriptive Cancer Epidemiology, Nagoya University Graduate School of Medicine, Nagoya, Aichi Japan; 9 Division of Cohort Research, National Cancer Center Institute for Cancer Control, Tokyo, Japan; 10 Department of Health Services Research, Faculty of Medicine, University of Tsukuba, Tsukuba, Ibaraki Japan; 11 Faculty of Epidemiology and Population Health, London School of Hygiene and Tropical Medicine, London, UK; 12 NCD Epidemiology Research Center, Shiga University of Medical Science, Otsu, Shiga Japan; 13 Department of Epidemiology for Community Health and Medicine, Graduate School of Medical Science, Kyoto Prefectural University of Medicine, Kyoto, Japan; 14 Department of Preventive Medicine, Institute of Biomedical Sciences, Tokushima University Graduate School, Tokushima, Japan; 15 Department of Comprehensive General Internal Medicine, Faculty of Medical Sciences, Kyushu University, Fukuoka, Japan; 16 Department of Preventive Medicine, Faculty of Medicine, Saga University, Saga, Japan; 17 Department of International Island and Community Medicine, Kagoshima University Graduate School of Medical and Dental Sciences, Kagoshima, Japan; 18 Aichi Cancer Center Research Institute, Nagoya, Aichi Japan; 19 Core Facilities, Aichi Cancer Center Research Institute, Nagoya, Aichi Japan; 20 Laboratory for Genotyping Development, RIKEN Center for Integrative Medical Sciences, Yokohama, Kanagawa Japan; 21 Department of Preventive Medicine, Nagoya University Graduate School of Medicine, Nagoya, Aichi Japan; 22 Department of Integrative Genomics, Tohoku Medical Megabank Organization, Tohoku University, Sendai, Miyagi Japan; 23 Department of Preventive Medicine and Epidemiology, Tohoku Medical Megabank Organization, Tohoku University, Sendai, Miyagi Japan; 24 Division of Clinical Research and Epidemiology, Iwate Tohoku Medical Megabank Organization, Iwate Medical University, Morioka, Iwate Japan; 25 Division of Cancer Epidemiology, Nagoya University Graduate School of Medicine, Nagoya, Aichi Japan; 26 National Institute of Health and Nutrition, National Institutes of Biomedical Innovation, Health and Nutrition, Tokyo, Japan; 27 Iwate Tohoku Medical Megabank Organization, Iwate Medical University, Morioka, Iwate Japan; 28 Tohoku Medical Megabank Organization, Tohoku University, Sendai, Miyagi Japan

**Keywords:** Cancer, Gastrointestinal diseases, Gastrointestinal cancer, Colorectal cancer

## Abstract

Observational studies suggest that abnormal glucose metabolism and insulin resistance contribute to colorectal cancer; however, the causal association remains unknown, particularly in Asian populations. A two-sample Mendelian randomisation analysis was performed to determine the causal association between genetic variants associated with elevated fasting glucose, haemoglobin A1c (HbA1c), and fasting C-peptide and colorectal cancer risk. In the single nucleotide polymorphism (SNP)-exposure analysis, we meta-analysed study-level genome-wide associations of fasting glucose (~ 17,289 individuals), HbA1c (~ 52,802 individuals), and fasting C-peptide (1,666 individuals) levels from the Japanese Consortium of Genetic Epidemiology studies. The odds ratios of colorectal cancer were 1.01 (95% confidence interval [CI], 0.99–1.04, *P* = 0.34) for fasting glucose (per 1 mg/dL increment), 1.02 (95% CI, 0.60–1.73, *P* = 0.95) for HbA1c (per 1% increment), and 1.47 (95% CI, 0.97–2.24, *P* = 0.06) for fasting C-peptide (per 1 log increment). Sensitivity analyses, including Mendelian randomisation-Egger and weighted-median approaches, revealed no significant association between glycaemic characteristics and colorectal cancer (*P* > 0.20). In this study, genetically predicted glycaemic characteristics were not significantly related to colorectal cancer risk. The potential association between insulin resistance and colorectal cancer should be validated in further studies.

## Introduction

Colorectal cancer comprises a heterogeneous group of neoplasms influenced by a variety of environmental factors and genes^1^. The drastic alteration in diets and lifestyle due to industrialisation and economic growth has led to an increased colorectal cancer incidence in the Asian population^2^. In fact, nearly a half of the newly diagnosed and prevalent colorectal cancer cases in the last five years have occurred in Asia^3^. Therefore, identifying modifiable risk factors is essential for reducing the incidence of the disease and the associated socioeconomic losses.

The pivotal role of impaired glucose tolerance and insulin resistance has been increasingly recognised in colorectal carcinogenesis^4–7^. Hyperglycaemia promotes glucose oxidation in intracellular mitochondria, and subsequent oxidative stress leads to DNA damage^6^. It is likely that proliferative and anti-apoptotic effects of insulin also promote colorectal tumour growth^4^. However, there are only a few epidemiological studies assessing the association between hyperglycaemia, hyperinsulinemia, or insulin-related traits and colorectal cancer, and the results remain inconclusive in Asians^8–12^. Although some observational studies suggest that metformin (a widely used hypoglycaemic agent) is associated with reduced colorectal cancer risk and cancer-specific mortality^13^, large-scale randomised controlled trials have not reported such a relationship^14^. Conflicting results also exist on the effect of thiazolidinediones (another type of glucose-lowering medication) on colorectal cancer^15,16^. These inconclusive results suggest that findings from observational studies may be distorted by confounding factors or reverse causation. Therefore, the causality between dysregulated glucose metabolism and colorectal cancer remains largely elusive.

Mendelian randomisation (MR) could overcome such biases^17^. MR uses genetic variants as instrumental variables to determine the unconfounded influence of exposure (glucose intolerance) on outcome (colorectal cancer). However, only a handful of MR studies have assessed the causality between glycaemic characteristics and colorectal cancer risk^18,19^, and none of these were from Asian populations. An MR study from the USA using an individual-level genome-wide association study (GWAS; n = 736 cases and 10,342 controls) did not find any significant associations between insulin-related traits and incident colorectal cancer^18^. More recently, a relatively large-scale MR study from the UK Biobank (n = 5,486 cases and 292,606 controls) reported that genetically predicted fasting glucose and fasting insulin were not significantly associated with colorectal cancer risk^19^. Considering that genetic variants are heterogeneous in different ethnicities that may presumably affect the susceptibility of disease^20^, an MR study in Asian populations is warranted.

Hence, in the present study, we leveraged large-scale Japanese samples including 7,936 colorectal cancer cases and performed an MR analysis to examine the association between genetically predicted measurements of hyperglycaemia (fasting glucose and HbA1c) and insulin resistance (fasting C-peptide) and colorectal cancer risk.

## Materials and methods

### Study design

The MR method estimates the relationship between the exposure and the outcome of interest using known genetic variants related to the exposure under the following assumptions: (i) the selected genetic instruments are associated with the exposure of interest; (ii) they are not associated with any confounding factors in the relationship between the exposure and the outcome; (iii) the association between the genetic instruments and the outcome is only through the exposure of interest^21^.

**Figure 1:**
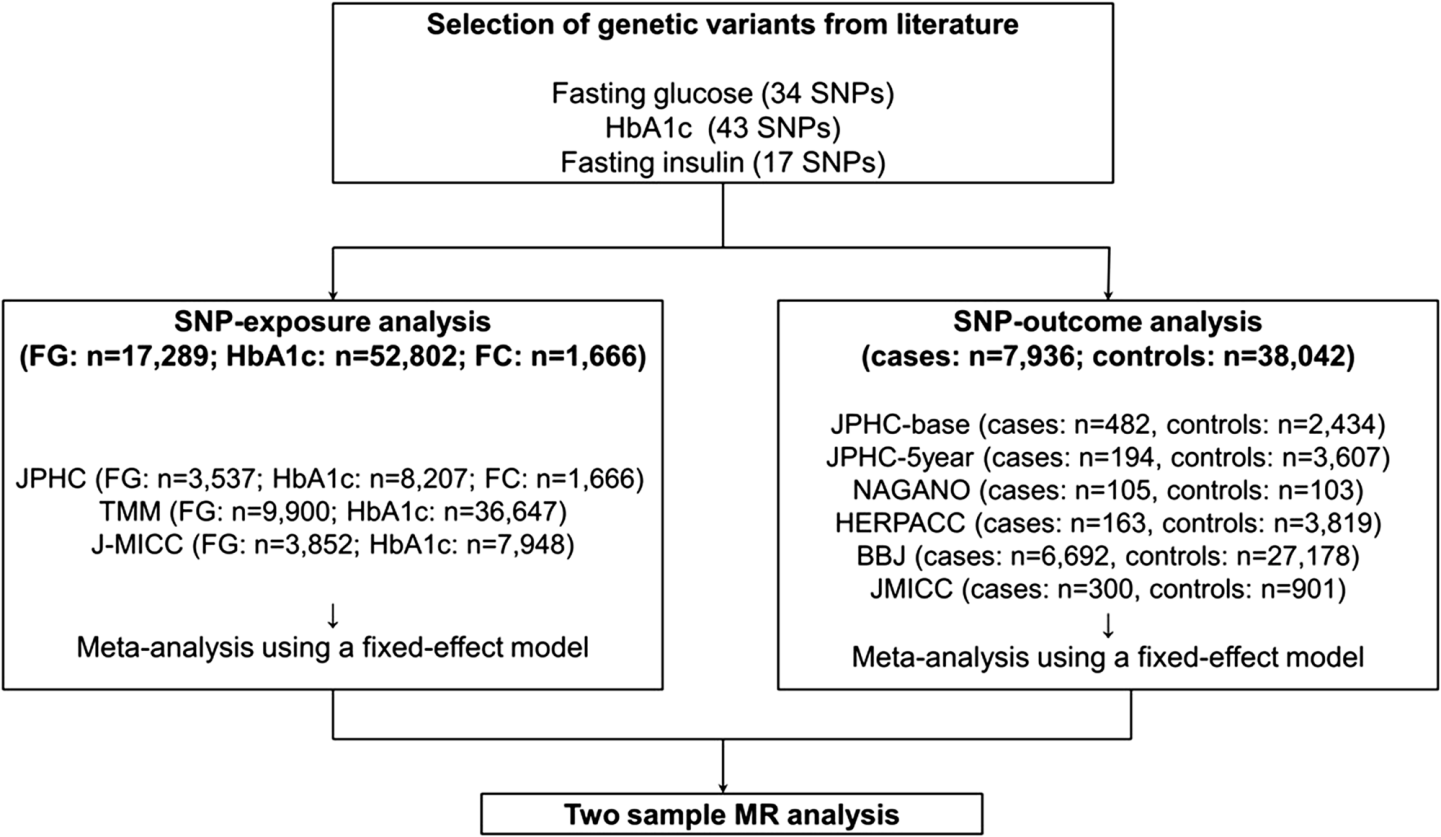
Mendelian randomisation study design. *BBJ* BioBank Japan, *FC* fasting C-peptide, *FG* fasting glucose, *FI* fasting insulin, *HbA1c* haemoglobin A1c, *HERPACC* the Hospital-based Epidemiologic Research Program at Aichi Cancer Centre, *J-MICC* the Japan Multi-Institutional Collaborative Cohort, *JPHC* Japan Public Health Center, *TMM* the Tohoku Medical Megabank, *SNP* single nucleotide polymorphism.

As shown in Fig. 1, we conducted two-sample MR analyses, in which we used two independent study samples to estimate the single nucleotide polymorphism (SNP)-risk factors (fasting glucose, HbA1c, and fasting C-peptide) and SNP-outcome (colorectal cancer) associations. There was a slight sample overlap between SNP-glycaemic traits and SNP-colorectal cancer analyses, which resulted in a bias toward non-null association^22^.

### Genetic instrument selection

To satisfy the MR assumption (i), instrumental variables for fasting glucose, HbA1c, and fasting C-peptide were systematically selected through previously published GWASs, as shown in Supplementary Figure S1 and Supplementary Table S1. Briefly, we used the GWAS Catalog (https://www.ebi.ac.uk/gwas/), co-published by the National Human Genome Research Institute and the European Bioinformatics Institute (for all three phenotypes). Given that GWASs for fasting C-peptide was extremely limited and none of the SNPs were met the baseline exclusion criteria (Supplementary Figure S1), we substituted fasting insulin for fasting C-peptide when searching SNPs through the GWAS Catalog. Genome-wide association data from the Meta-Analysis of Glucose and Insulin-related traits Consortium on 1.3 + billion Caucasian individuals free of diabetes were also used when selecting SNPs for fasting glucose and fasting insulin (substitute for fasting C-peptide)^23^. We selected SNPs for each glycaemic trait as instruments (genetic variants) reaching a genome-wide statistical significance threshold (*P* < 5 × 10^–8^), with minor allele frequency > 0.01 in the East Asians of the 1000 Genomes Project. As of December 1, 2020, we identified 97, 94, and 23 instruments for fasting glucose, HbA1c, and fasting C-peptide, respectively. To minimise the potential confounding effects from linkage disequilibrium, we used the “clumping” function in PLINK (a widely used open-source toolset for population-based linkage analyses and GWASs) (R^2^ > 0.001, with a 1 Mb window). Finally, we identified 34, 43, and 17 instruments for fasting glucose, HbA1c, and fasting C-peptide, respectively. Regardless of the statistical significance of the SNP-glycaemic trait associations in our samples, we used all the selected SNPs as instrumental variables to minimise biases from false negatives due to insufficient power^24^ and overfitting^25^. Detailed information for instrumental variables of each glycaemic trait is presented in Supplementary Tables S2 and S3.

Considering that several variants may affect HbA1c levels via erythrocyte biology^26^, thereby violating the MR assumptions (ii) and (iii), we examined whether the selected instruments are associated with erythrocyte-related traits at the GWAS significance threshold (*P* < 5 × 10^–8^) using the PhenoScanner (available at http://www.phenoscanner.medschl.cam.ac.uk/phenoscanner) and Haploreg databases (available at: http://archive.broadinstitute.org/mammals/haploreg/haploreg.php). The instruments included rs579459 for haemoglobin concentration; rs17509001 and rs13134327 for high light scatter reticulocyte count; rs6684514, rs7616006, rs4737009, rs12602486, and rs4820268 for mean corpuscular haemoglobin concentration; rs11248914, rs9914988, rs2748427, and rs57601949 for mean corpuscular volume; rs11964178, rs7776054, rs6980507, rs10823343, rs174594, and rs12819124 for red blood cell count; rs857691, rs9818758, rs837763, and rs17533903 for reticulocyte count; and rs282587 for reticulocyte fraction of red cells. Additionally, we examined whether selected SNPs, such as body mass index (BMI), smoking, alcohol intake, and physical inactivity, could be confounders of the association between glycaemic metabolism and colorectal cancer^2^. While some instruments were associated with these traits (for example, rs2237892 on *KCNQ* for BMI), manual selection of instrumental variables that may have pleiotropic effects is generally not recommended^27^. Therefore, we conducted an MR-Egger regression^28^ as a sensitivity analysis (details are described in the Statistical Analysis section).

### Proportion of explained variance and F-statistics

We calculated how much the selected genetic variant could explain the respective phenotype (*X*) using the previously described formula as below:

$${R}^{2}=\frac{{\sum }_{i=1}^{N} 2\left({p}_{i}\right)\left(1-{p}_{i}\right){\beta }_{i}^{2}}{Var(X)}$$

(*R*^*2*^ for the proportion of explained variance, *N* for the independent SNP instruments, ${p}_{i}$ for the effect allele frequency of *SNP*_*i*_ and ${\beta }_{i}$ for the magnitude of association between *SNP*_*i*_ and the phenotype)^29^.

Based on the explained variance for each glycaemic trait, we performed power calculations for MR analyses, setting a type-I error rate of 5% and power of 80%^29^. We also calculated the approximate F-statistics, which reflects the “strength” of an instrumental variables. The F-statistic can be approximated as

$${F}=\frac{{R}^{2} (n-1-k)}{(1-{R}^{2})k}$$

(*R*^*2*^ for the proportion of explained variance, *n* for the sample size, and *k* for the number of instrumental variables)^30^.

### Genetic associations with glycaemic traits (fasting glucose, HbA1c, and C-peptide)

We obtained the summary statistics of the SNP-glycaemic trait (fasting glucose and HbA1c) associations from the Japanese Consortium of Genetic Epidemiology (J-CGE) studies^31^, which consists of the Japan Public Health Center (JPHC)-based prospective study, the Tohoku Medical Megabank (TMM) Community-Based Cohort study, the Japan Multi-Institutional Collaborative Cohort (J-MICC) study, and the Hospital-based Epidemiologic Research Program at Aichi Cancer Center (HERPACC). In the current MR study, genetic data for each glycaemic trait were available from JPHC, TMM and J-MICC within the J-CGE studies. The characteristics of each cohort are presented in Table 1 and Supplementary Table S4. As a proxy for insulin resistance, we used the measurement of fasting C-peptide, which is a more stable measure of insulin^32^, from the JPHC study.

**Table 1:** Characteristics of the studies considered for the analysis of single nucleotide polymorphism-exposure associations in the Japanese Consortium of Genetic Epidemiology Studies.

Phenotype	Source	Participants	Mean ± SD	Units	No. of SNPs	Variance explained, % (F-statistic)	Population
Fasting glucose levels	JPHC	3,537 Non-diabetic individuals	94.3 ± 10.1	mg/dL	34	2.48 (13.71)	Japanese
	TMM	9,900 Non-diabetic individuals	88.2 ± 10.0				
	J-MICC	3,852 Non-diabetic individuals	94.4 ± 9.3				
HbA1c	JPHC	8,207 Non-diabetic individuals	5.3 ± 0.4	%	43	1.22 (15.15)	Japanese
	TMM	36,647 Non-diabetic individuals	5.5 ± 0.3				
	J-MICC	7,948 Non-diabetic individuals	5.4 ± 0.3				
Fasting C-peptide levels	JPHC	1,666 Non-diabetic individuals	1.4 ± 0.5	ng/mL	17	1.04 (1.08)	Japanese

*HbA1c* haemoglobin A1c, *HERPACC* the Hospital-based Epidemiologic Research Program at Aichi Cancer Center, *J-MICC* the Japan Multi-Institutional Collaborative Cohort, *JPHC* Japan Public Health Center, *TMM* the Tohoku Medical Megabank.

The exclusion criteria included: participants with physician-diagnosed diabetes; participants on any diabetes treatment; participants with fasting (defined by ≥ 8 h) serum glucose ≥ 126 mg/dL (≈ 7 mmol/L for fasting glucose or fasting C-peptide) or HbA1c ≥ 6.5% (for HbA1c); people with missing data on fasting status. In total, we included 17,289 people with fasting glucose measurements (n = 3,537 in JPHC; 9,900 in TMM; and 3,852 in J-MICC), 52,802 people with HbA1c (n = 8,207 in JPHC; 36,647 in TMM; and 7,948 in J-MICC), and 1,666 participants with fasting C-peptides (n = 1,666 in JPHC). The details of each GWAS are described in Supplementary Table S5 For fasting glucose and HbA1c, we meta-analysed each β coefficient and its 95% confidence interval (CI) from the individual study and further meta-analysed the overall effects of SNP-exposure in the fixed-effects inverse-variance weighted (IVW) method. Fasting glucose (mg/dL) and HbA1c (%) were unchanged. Fasting C-peptide (ng/mL) was log-transformed to enhance compliance with normality.

### Genetic associations with colorectal cancer

Data for colorectal cancer were extracted from the individual-level GWAS data, including the JPHC case-cohort study-base (482 colorectal cancer cases and 2,434 control subjects), the JPHC case-cohort study-5 years (194 colorectal cancer cases and 3,607 controls), NAGANO study (105 colorectal cancer cases and 103 control subjects), HERPACC study (163 colorectal cancer cases and 3,819 control subjects), and J-MICC study (300 colorectal cancer cases and 901 control subjects); further, summary-level GWAS data were extracted from the BioBank Japan (BBJ) study (6,692 colorectal cancer cases and 27,178 control subjects; NDBC with the primary accession code hum0014; available at https://humandbs.biosciencedbc.jp/hum0014-v18^33^(Supplementary Table S6). The GWAS used phase 1 (for the BBJ study) and phase 3 (for the other institutions) of the 1000 Genomes Project as a reference panel in the imputation stage with adjustment of genetic principal components (Supplementary Table S5). The overall estimates of the SNP outcomes were combined using an IVW meta-analysis. This study was approved by the review board of the National Cancer Centre, Japan (Approval No.: 2011-044), TMM (Approval No.: 2012-4-617), Iwate Medical University (HG H25-2), Aichi Cancer Center (Approval No.: 12-27), and the Nagoya University Graduate School of Medicine (Approval No.: 2010-0939). Participants in the JPHC, who had provided blood, were contacted by mail and given the opportunity to opt out of participation before initiating this study. In addition, information on the study was posted on the website of the JPHC to provide participants with the opportunity to opt-out at any time of which the protocol was approved by the institutional review board of the National Cancer Center. Written informed consent was obtained from all the participants in the rest of the institutions. Details are shown in Supplementary Table S4. All procedures contributing to this study comply with the ethical standards of the relevant institutional committees on research involving human participants and with the Helsinki Declaration of 1964, as revised in 2008.

### Statistical analysis

We used the TwoSampleMR package in R v3.6.4 for MR analyses, excluding the MR-Pleiotropy Residual Sum and Outlier (MR-PRESSO) model, which utilised the MR-PRESSO package v1.0. The IVW method with random effects was used to assess the relationship between genetically predicted glycaemic traits (fasting glucose, HbA1c, and fasting C-peptide) and the risk of colorectal cancer.

While the IVW method has great statistical power, this method requires a stringent assumption; all the instrumental variables are valid or “balanced pleiotropy”^34^. In the presence of directional horizontal pleiotropy, however, the IVW method may produce biased estimates^35^. To address such violations (MR assumptions (ii) and (iii)), we further applied multiple sensitivity analyses that are more robust to pleiotropic effects, including MR-Egger regression, weighted median, and MR-PRESSO analyses. The intercept term of the MR-Egger regression provides the indicator of unbalanced pleiotropy (*P* < 0.05 indicated significance)^28^. Nonetheless, the disadvantage of the MR-Egger approach is that it is affected by outliers or influential data points. At this point, the weighted-median analysis is useful because the approach can produce valid estimates if at least a half of the instruments are correct^35^. Finally, the MR-PRESSO method performs regression analysis of the estimates for SNP-outcome against the SNP-exposure to explore outlier SNPs^36^. Outliers were then removed from genetic variants whose causal estimates differ substantially from those of the other variants, and the IVW method for all variants was then performed. The funnel and leave-one-out plots were also obtained. The thresholds for nominal significance were set at *P* < 0.05.

## Results

### Participant characteristics

For the SNP-exposure analyses, we used participants from the J-CGE studies, as shown in Table 1. The mean age of patients in each cohort ranged from 54.0 to 63.0 years, and the proportion of women was 46.1–69.8% (Supplementary Table S7). The mean value of each glycaemic trait was 88.2–94.4 mg/dL for fasting glucose, 5.3–5.5% for HbA1c, and 1.4 ng/mL for fasting C-peptide (Table 1). For the SNP-outcome analyses, Supplementary Table S8 presents the characteristics of the participants (n = 7,936 colorectal cancer cases and 38,042 controls) from the six studies (JPHC case-cohort study-base, JPHC case-cohort study-5 years, NAGANO, HERPACC, BBJ, and J-MICC). The mean age and the proportion of women were 52.1–59.3 years and 37.0–62.3%, respectively (Supplementary Table S8). The explained variance of genetic variants (F-statistics) used as genetic instruments was 2.48% (13.71) for fasting glucose, 1.22% (15.15) for HbA1c, and 1.04% (1.08) for fasting C-peptide (Table 1). Based on the calculated explained variance, the current study (n = 7,936 cases and 38,042 controls) identified odds ratios (ORs) of ≥ 1.02 (per 1 mg/dL) for fasting glucose, ≥ 2.04 (per 1%) for HbA1c, and ≥ 1.98 (per 1 log) for fasting C-peptide, indicating a statistically significant association.

### Mendelian randomisation analysis

In the primary IVW MR analyses, the genetic predisposition to fasting glucose and HbA1c levels were not significantly associated with the risk of colorectal cancer. The respective estimates of OR (95% CI) for colorectal cancer were 1.01 (0.99–1.04, *P* = 0.34) for fasting glucose (per 1 mg/dL increment) and 1.02 (0.60–1.73, *P* = 0.95) for HbA1c (per 1% increment) (Table 2 and Supplementary Figures S2, S3). We found a suggestive association between the genetic predisposition to fasting C-peptide and colorectal cancer risk (the central estimate of OR [95% CI] for 1 log increment = 1.47 [0.97–2.24], *P* = 0.06) (Table 2 and Supplementary Figure S4). However, in sensitivity analyses, including the weighted-median and MR-Egger approaches, a suggestive association between fasting C-peptide and colorectal cancer risk was not observed (Table 2). Similar to the IVW MR results, there was no association between fasting glucose and HbA1c levels with colorectal cancer risk in all sensitivity analyses (Table 2). While the funnel plot showed some asymmetrical distribution (Supplementary Figures S2–S4), the *P* value for the MR-Egger intercept was not statistically significant (0.12 for fasting glucose, 0.23 for HbA1c, and 0.56 for C-peptides), suggesting no pleiotropic effect (Table 2). In the MR-PRESSO approach, one SNP for both fasting glucose and HbA1c was excluded, while no outlier was found for fasting C-peptide. Additionally, the leave-one-out analysis assessing the impact of a potential outlier SNP showed similar results (Supplementary Figures S2–S4).

**Table 2:** Mendelian randomisation analysis of glycaemic traits with colorectal cancer risk.

Phenotype	OR (95% CI)	*P* value
Fasting glucose levels (mg/dL)		
IVW-random effects	1.01 (0.99–1.04)	0.34
MR Egger (P for pleiotropy = 0.12)	0.98 (0.93–1.03)	0.37
Weighted median	1.00 (0.98–1.03)	0.86
MR-PRESSO^†^	1.00 (0.99–1.02)	0.71
HbA1c (%)		
IVW-random effects	1.02 (0.60–1.73)	0.95
MR Egger (P for pleiotropy = 0.23)	0.58 (0.20–1.66)	0.31
Weighted median	0.74 (0.41–1.34)	0.29
MR-PRESSO^†^	0.89 (0.58–1.37)	0.93
Fasting C-peptide levels (ln ng/mL)		
IVW-random effects	1.47 (0.97–2.24)	0.06
MR Egger (P for pleiotropy = 0.56)	1.19 (0.53–2.70)	0.68
Weighted median	1.16 (0.59–2.26)	0.67
MR-PRESSO	1.47 (0.97–2.24)	0.06

^**†**^One outlier was detected and corrected in the MR-PRESSO analyses for fasting glucose and HbA1c.

*CI* confidence interval, *HbA1c* haemoglobin A1c, *IVW* inverse-variance weighted, *MR* Mendelian randomisation, *PRESSO* Pleiotropy Residual Sum and Outlier, *OR* odds ratio.

## Discussion

Utilising the genetic instrumental variables in the association between various glycaemic traits and colorectal cancer, we performed a large-scale MR analysis in the Japanese populations. There was no strong evidence for a relationship between fasting glucose and HbA1c levels and colorectal cancer. While we observed potential positive association between higher fasting C-peptide and colorectal cancer in our primary analysis using the IVW MR method, we did not observe this based on the MR-Egger or the weighted-median approach. However, since this is the first MR study to comprehensively evaluate the association of glycaemic traits with colorectal cancer in Asian populations, our findings need to be validated further.

The present MR findings are inconsistent with the observational studies that suggest a positive link between hyperglycaemia and colorectal cancer risk^5,12^. A meta-analysis from 18 studies showed a dose–response positive relation between fasting glucose levels and colorectal cancer across different study designs (case–control vs. cohort studies) and ethnicities (Europeans vs. Asians)^5^. Likewise, several cohort studies identified an elevated risk of colorectal cancer with higher HbA1c^37^, although two nested case–control studies from the USA found no association (presumably because case–control designs were extremely sensitive for selection bias and reverse causation)^38,39^. Furthermore, in an umbrella review of 30 observational studies, including 62,163 cases, colorectal cancer is one of the four cancer types associated with diabetes^7^. In contrast, our null findings were generally in line with recently published MR studies^18,19^. A study with 5,486 cases among 367,643 patient samples of European descent from the UK Biobank, which used 35 instrumental variables, reported an insignificant association of fasting glucose with colorectal cancer^19^. Using four SNPs, another individual-level MR study with 11,078 non-Hispanic white postmenopausal women, including 736 cases, found null associations between fasting glucose and colorectal cancer^18^. Effect estimates of MR meta-analysis for fasting glucose and colorectal cancer were 1.09 (ranging from 0.95 to 1.24; *P* = 0.212)^19^. Hence, these findings provide limited evidence of a genetic role of hyperglycaemia per se in colorectal carcinogenesis.

Epidemiological evidence for the association of insulin and insulin-related traits with incident colorectal cancer has been inconclusive, particularly among Asians^8–11^. For instance, several case–control studies^9,10^ and a cohort study^11^ over a 20-year follow-up period showed a non-significant relation between fasting insulin levels and colorectal cancer. In contrast, a nested case–control study (n = 375 colorectal cancer cases among 38,373 adults)^8^ from Japan suggested a significant positive association between plasma C-peptide and colorectal cancer in men (OR [95% CI] comparing highest vs. lowest quartiles of C-peptide = 3.2 [1.4–7.6]). A recent meta-analysis of 27 studies with over 10,000 cases of colorectal adenomas (which are precancerous and thus considered as an important indicator of colorectal tumorigenesis)^40^ suggested a stronger positive association of insulin-related traits in non-Asians (summary OR [95% CI] for insulin = 1.67 [1.28–2.17] and for C-peptide = 1.59 [1.22–2.08]) than in Asians (summary OR [95% CI] for insulin = 1.10 [0.92–1.33] and for C-peptide = 1.27 [0.92–1.91])^41^. Given that Asians are more prone to insulin resistance for a given BMI than Caucasians^42^, the observed heterogeneity could be partially explained by the population difference or inherent biases of residual confounding and reverse causality. Collectively, we have conducted an MR study that is less susceptible to such biases because alleles are randomly assigned in meiosis and unaffected by the acquired factors^21^.

We found suggestive evidence that genetic predisposition to insulin resistance (fasting C-peptide) may influence colorectal cancer risk in Asians in our primary analysis using the IVW MR method. However, the CI was too wide to make a definitive conclusion. Among several biomarkers indicating insulin secretion, C-peptide is a relatively stable biomarker with less sensitivity to fasting status and a longer circulating half-life^32^, adding to the robustness of the estimates. Nonetheless, our sensitivity analyses, which were less sensitive to invalid genetic instruments, did not support this association. The two MR studies in non-Asians also did not show strong evidence for the insulin-colorectal cancer axis^18,19^. Non-significant findings from MR studies, including this study, might also arise from insufficient power. Indeed, recent large-scale MR studies supported a significant association between fasting insulin and colorectal cancer risk^43,44^. Other factors may include the diverse genetic instruments for insulin secretion, which might be associated with different colorectal cancer pathways^34^. For instance, among the selected instrumental variables, rs8050136 at the *FTO*^22^ might affect increased BMI^45^, thereby contributing to colorectal carcinogenesis^31^ partly via hyperinsulinemia, since obesity is a risk factor for increased insulin secretion^46^. This type of pleiotropy, known as vertical pleiotropy, is not a barrier in the causal inference of MR studies^27^. In contrast, some genetic variants could be associated with colorectal cancer through unknown pathways unrelated to insulin resistance (horizontal pleiotropy). Nonetheless, we conducted multiple sensitivity analyses, including MR-PRESSO and Egger regression, suggesting no pleiotropic effect in our current study.

Experimental and clinical evidence supports a role for insulin and insulin-like growth factor (IGF) signalling systems in colorectal carcinogenesis^47^. Although insulin and IGF1 receptors are both expressed in normal and cancerous colorectal epithelia, their bioactivities are slightly different^48^. IGFs play a role in cell proliferation, whereas insulin is responsible for carbohydrate metabolism^48^. Since IGF1 can directly promote cellular proliferation through the Ras/MAPK (mitogen-activated protein kinase) and PI3K (phosphoinositide 3-kinase)-Akt (protein kinase B) pathways, the mitogenic properties of insulin may be mediated via IGF1 receptors or its hybrids^48^. The significant role of IGFs in colorectal tumorigenesis is supported by a recent MR study from the UK Biobank, including 52,865 colorectal cancer cases^49^. Although further studies are warranted, dysregulated insulin pathways, rather than a consequence of impaired glucose metabolism, might have a higher chance of increasing colorectal cancer risk.

This is the first MR study that assessed the causality of glycaemic traits and colorectal cancer among Asian populations on a relatively large scale. The current MR framework can diminish confounding and reverse causality and potentially bias the observational findings. However, a limitation of our analysis included measurement errors, since all biomarkers were measured only once at the baseline survey. Due to the limited number of GWASs from East Asian populations, we have systematically identified the SNPs from publicly available GWASs in all ethnicities. Therefore, we cannot eliminate the possibility that some SNPs were not related to glycaemic traits in East Asians, which may lead to bias. Nonetheless, we have restricted the SNPs with minor allele frequency > 0.01 in East Asians as genetic instrumental variables and thus such bias should be small, if existent. In addition, for the sensitivity analyses, we only used the SNPs from the GWASs conducted in East Asians and confirmed that the MR results were generally comparable. For SNP-exposure analyses, the sample size was limited because we only included subjects with fasting status over 8 h after a meal, leading to the low precision of the exposure measurement; however, the explained variance in this study (2.48% for fasting glucose and 1.04% for fasting C-peptide) was generally comparable to that reported in previous studies (approximately ~ 5% for fasting glucose, 1% for fasting insulin)^19,23,50,51^. Nonetheless, we admit that F-statistics, whose threshold of < 10 defines a “weak instrumental variable” in the MR study^30^, was limited to provide robust estimates for fasting C-peptide (F-statistic for fasting C-peptide: 1.08) and thus the current results should be interpreted cautiously. In addition to the limited explained variance for each glycaemic trait and the limited number of colorectal cancer cases, weak associations could potentially remain undetected. In particular, an observed marginal association between fasting C-peptide and colorectal cancer risk may be due to the limited power or due to the weak instrument bias. Therefore, we cannot eliminate a possibility that the observed marginal association may reach a statistically significant or may become totally insignificant when a sample size of colorectal cancer cases increases. In addition, subgroup analyses by disease aetiology should also require larger sample sizes. Therefore, further studies with sufficient sample sizes are warranted. Finally, there was a sample overlap between the exposure and the outcome datasets, leading to a non-null association^22^. Hence, the observed marginal positive association between fasting C-peptide and colorectal cancer risk might be overestimated. However, owing to the small proportion of such cases, the bias should be minimal.

In summary, our findings provide no strong evidence to support an association between hyperglycaemia and colorectal cancer among Asian populations; however, a possible association between insulin resistance and colorectal cancer warrants further study with a larger sample size.

## Supplementary Information

Supplementary Information.

## Data Availability

All materials used in this MR study (i.e., effect estimates and standard errors of single nucleotide polymorphisms used as instrumental variables) are available in the Supplementary materials.
