# Supplementary material for: Investigating the association between glycaemic traits and colorectal cancer in the Japanese population using Mendelian randomisation
Source: Sci Rep. 2023 Apr 29;13:7052. doi: 10.1038/s41598-023-33966-7 (PMC10148817; doi:10.1038/s41598-023-33966-7)
Supplement: Supplementary file 1 — Supplementary Information. [file 41598_2023_33966_MOESM1_ESM.docx]

**Investigating the association between glycaemic traits and colorectal cancer in the Japanese population using Mendelian randomisation**

Akiko Hanyuda, Atsushi Goto, Ryoko Katagiri, Yuriko N. Koyanagi, Masahiro Nakatochi, Yoichi Sutoh, Shiori Nakano, Isao Oze, Hidemi Ito, Taiki Yamaji, Norie Sawada, Masao Iwagami, Aya Kadota, Teruhide Koyama, Sakurako Katsuura-Kamano, Hiroaki Ikezaki, Keitaro Tanaka, Toshiro Takezaki, Issei Imoto, Midori Suzuki, Yukihide Momozawa, Kenji Takeuchi, Akira Narita, Atsushi Hozawa, Kengo Kinoshita, Atsushi Shimizu, Kozo Tanno, Keitaro Matsuo, Shoichiro Tsugane, Kenji Wakai, Makoto Sasaki, Masayuki Yamamoto, Motoki Iwasaki

**Supplementary materials**

Supplementary Table S1. Studies selected from the GWAS Catalog

Supplementary Table S2. Single nucleotide polymorphisms used as instrumental variables for fasting glucose, HbA1c, and fasting C-peptide

Supplementary Table S3. Single nucleotide polymorphisms used as instrumental variables for colorectal cancer

Supplementary Table S4. Study description in terms of exposure (fasting glucose, HbA1c, and fasting C-peptide)

Supplementary Table S5. Information on genotyping methods, quality controls for single nucleotide polymorphisms, imputation, and statistical analysis for studies

Supplementary Table S6. Study description in terms of outcome (colorectal cancer)

Supplementary Table S7. Characteristics of the studies considered for the analysis of SNP-glycaemic trait associations

Supplementary Table S8. Characteristics of the studies considered for the analysis of SNP-colorectal cancer associations

Supplementary Figure S1. Flow chart of single nucleotide polymorphism selection from the literature

Supplementary Figure S2. Analyses of fasting glucose and colorectal cancer risk

a). Scatter plots of genetic association with colorectal cancer (outcome) over genetic association with fasting glucose (exposure)

b). Leave-one-out sensitivity analysis of genetic association with colorectal cancer (outcome) over genetic association with fasting glucose (exposure)

c). Funnel plot of IV precisions (1/SE_IV_) against the IV estimates (β_IV_) for fasting glucose

Supplementary Figure S3. Analyses of HbA1c and colorectal cancer risk

a). Scatter plots of genetic association with colorectal cancer (outcome) over genetic association with HbA1c (exposure)

b). Leave-one-out sensitivity analysis of genetic association with colorectal cancer (outcome) over genetic association with HbA1c (exposure)

c). Funnel plot of IV precisions (1/SE_IV_) against the IV estimates (β_IV_) for HbA1c

Supplementary Figure S4. Analyses of fasting C-peptide and colorectal cancer risk

a). Scatter plots of genetic association with colorectal cancer (outcome) over genetic association with fasting C-peptide (exposure)

b). Leave-one-out sensitivity analysis of genetic association with colorectal cancer (outcome) over genetic association with fasting C-peptide (exposure)

c). Funnel plot of IV precisions (1/SE_IV_) against the IV estimates (β_IV_) for fasting C-peptide

**Supplementary Table S1. Studies selected from the GWAS Catalog**

| **Phenotype** | **Publication** | **Populations** | **Sample size** | **Number of SNPs** | **First author** | **Journal** | **Publication date** |
| --- | --- | --- | --- | --- | --- | --- | --- |
| Fasting glucose | 17903298 | NR | 1,027 individuals | 1 | Meigs JB | BMC Med Genet | 2007/9/19 |
|  | 20081858 | Europeans | 100,502 individuals | 9 | Dupuis J | Nat Genet | 2010/1/17 |
|  | 22581228 | Europeans | 85,573 individuals | 3 | Manning AK | Nat Genet | 2012/5/13 |
|  | 22885924 | Europeans | 133,010 non-diabetic individuals | 11 | Scott RA | Nat Genet | 2012/8/12 |
|  | 25187374 | East Asians | 46,085 individuals | 5 | Hwang JY | Diabetes | 2014/9/3 |
|  | 25631608 | Trans-ethnic | 50,900 European ancestry non-diabetic individuals,  9,664 African American non-diabetic individuals | 1 | Wessel J | Nat Commun | 2015/1/29 |
|  | 28270201 | Europeans | 19,965 individuals | 1 | Nagy R | Genome Med | 2017/3/7 |
|  | 31021400 | Europeans | 46,186 individuals | 1 | Masotti M | Biometrics | 2019/4/25 |
|  | 31170924 | Koreans | 9,932 individuals | 1 | Kong S | BMC Med Genet | 2019/6/6 |
|  | 31217584 | Trans-ethnic | 9,720 African American individuals, 17,373 Hispanic/Latino individuals, 2,316 Asian ancestry individuals, 1,900 Native Hawaiian ancestry individuals, 591 Native American ancestry individuals, 321 individuals | 1 | Wojcik GL | Nature | 2019/6/19 |
| HbA1c | 24647736 | Asians | 17,290 East Asian ancestry individuals, 1,727 Malay ancestry individuals, 13585 East Asian individuals | 2 | Chen P | Diabetes | 2014/3/19 |
|  | 28887542 | Europeans | 35623 Europeans | 1 | Prins BP | Sci Rep | 2017/9/8 |
|  | 28898252 | South Asians | 7,572 South Asians | 23 | Wheeler E | PLoS Med | 2017/9/12 |
|  | 29403010 | Japanese | 42,790 Japanese individuals | 17 | Kanai M | Nat Genet | 2018/2/5 |
| Fasting C-peptide* | 20081858 | Europeans | 100,502 | 1 | Dupuis J | Nat Genet | 2010/1/17 |
|  | 22581228 | Europeans | 85,573 | 2 | Manning AK | Nat Genet | 2012/5/13 |
|  | 22885924 | Europeans | 108,557 non-diabetic individuals | 10 | Scott RA | Nat Genet | 2012/8/12 |
|  | 28898252 | South Asians | 7,572 South Asians | 1 | Wheeler E | PLoS Med | 2017/9/12 |
|  | 31217584 | Trans-ethnic | 9,720 African American individuals, 17,373 Hispanic/Latino individuals, 2,316 Asian ancestry individuals, 1,900 Native Hawaiian ancestry individuals, 591 Native American ancestry individuals, 321 individuals | 3 | Wojcik GL | Nature | 2019/6/19 |

*Due to the unavailability of selecting SNPs for FC, we substituted fasting insulin for FC when selecting instrumental variables.

Abbreviations: HbA1c, haemoglobin A1c.

**Supplementary Table S2. Single nucleotide polymorphisms used as instrumental variables for fasting glucose, HbA1c, and fasting C-peptide**

| **Trait** | **Nearby gene** | **Chr** | **Chr position (hg19)** | **rs ID** | **REF** | **ALT** | **Effect** | **SE** | **P-value** | **EAF** | **I^2^** | **q-pvalue**^†^ |
| --- | --- | --- | --- | --- | --- | --- | --- | --- | --- | --- | --- | --- |
| FG | *PROX1* | 1 | 214159256 | rs340874 | T | C | 0.219206 | 0.101255 | 3.04×10^-2^ | 0.391485 | 0 | 0.5438 |
| FG | *KRTCAP2P1, SIX3* | 2 | 45188353 | rs895636 | C | T | 0.783856 | 0.099623 | 3.60×10^-15^ | 0.421699 | 0 | 0.6913 |
| FG | *GCKR* | 2 | 27741237 | rs780094 | T | C | 0.852007 | 0.099789 | 1.37×10^-17^ | 0.443599 | 0 | 0.4331 |
| FG | *G6PC2* | 2 | 169763148 | rs560887 | T | C | 0.705426 | 0.32061 | 2.78×10^-2^ | 0.974460 | 42 | 0.1783 |
| FG | *DPYSL5* | 2 | 27152874 | rs1371614 | C | T | 0.227719 | 0.136922 | 9.63×10^-2^ | 0.153309 | 0 | 0.8472 |
| FG | *SLC2A2* | 3 | 170713290 | rs1280 | T | C | 0.025754 | 0.341432 | 9.40×10^-2^ | 0.021026 | 0 | 0.6037 |
| FG | *IGF2BP2* | 3 | 185513392 | rs7651090 | A | G | 0.186265 | 0.106662 | 8.08×10^-2^ | 0.308554 | 0 | 0.8642 |
| FG | *ZBED3-AS1* | 5 | 76425867 | rs7708285 | G | A | 0.018747 | 0.367308 | 9.59×10^-2^ | 0.982870 | 0 | 0.844 |
| FG | *MIR583HG* | 5 | 95539448 | rs4869272 | C | T | 0.279131 | 0.117397 | 1.74×10^-2^ | 0.768765 | 57.1 | 0.0972 |
| FG | *CDKAL1* | 6 | 20685486 | rs9356744 | T | C | 0.499253 | 0.100795 | 7.30×10^-7^ | 0.414930 | 40.1 | 0.1883 |
| FG | *RREB1* | 6 | 7213200 | rs17762454 | C | T | 0.097844 | 0.102161 | 3.38×10^-2^ | 0.367189 | 47.4 | 0.1493 |
| FG | *GTF3AP5* | 7 | 15064309 | rs2191349 | G | T | 0.795411 | 0.10941 | 3.59×10^-13^ | 0.670149 | 21.7 | 0.2789 |
| FG | *YKT6* | 7 | 44245853 | rs917793 | A | T | 0.765311 | 0.122253 | 3.85×10^-10^ | 0.201035 | 34.1 | 0.2191 |
| FG | *ZMAT4* | 8 | 40484239 | rs2722425 | T | C | -0.18227 | 0.110742 | 9.98×10^-2^ | 0.727011 | 41.6 | 0.1802 |
| FG | *RNU6-526P, RNU6-1151P* | 8 | 9177732 | rs983309 | T | G | -1.52082 | 0.466519 | 1.11×10^-3^ | 0.977364 | 9.1 | 0.333 |
| FG | *SLC30A8* | 8 | 118185733 | rs11558471 | A | G | -0.57015 | 0.098911 | 8.20×10^-9^ | 0.449801 | 21.8 | 0.2786 |
| FG | *CDKN2B-AS1* | 9 | 22134094 | rs10811661 | T | C | -0.53487 | 0.099881 | 8.55×10^-8^ | 0.437637 | 11.2 | 0.3244 |
| FG | *GLIS3* | 9 | 4293150 | rs10814916 | A | C | 0.228968 | 0.102991 | 2.62×10^-2^ | 0.458665 | 0 | 0.4727 |
| FG | *KANK1* | 9 | 622523 | rs10815355 | G | T | 0.422827 | 0.155652 | 6.60×10^-3^ | 0.142533 | 0 | 0.9904 |
| FG | *DNLZ* | 9 | 139256766 | rs3829109 | G | A | -0.34261 | 0.144251 | 1.76×10^-2^ | 0.139511 | 0 | 0.6534 |
| FG | *BTBD7P2* | 10 | 113042093 | rs10885122 | T | G | 0.305823 | 0.167322 | 6.76×10^-2^ | 0.901746 | 83.7 | 0.002202 |
| FG | *TCF7L2* | 10 | 114754088 | rs7901695 | T | C | 0.600784 | 0.23533 | 1.07×10^-2^ | 0.045795 | 0 | 0.6668 |
| FG | *FADS1* | 11 | 61571478 | rs174550 | T | C | -0.37234 | 0.099902 | 1.94×10^-4^ | 0.417274 | 0 | 0.8006 |
| FG | *ARAP1* | 11 | 72432985 | rs11603334 | G | A | -0.55186 | 0.269613 | 4.07×10^-2^ | 0.036191 | 0 | 0.9705 |
| FG | *MTNR1B* | 11 | 92708710 | rs10830963 | C | G | 0.599059 | 0.100752 | 2.75×10^-9^ | 0.417156 | 6.9 | 0.3417 |
| FG | *MADD* | 11 | 47346723 | rs11039182 | T | C | -0.41529 | 0.449181 | 3.55×10^-1^ | 0.013595 | 55.7 | 0.1046 |
| FG | *GLS2* | 12 | 56865338 | rs2657879 | A | G | 0.167869 | 0.162102 | 3.00×10^-1^ | 0.103048 | 18.3 | 0.2942 |
| FG | *PDX1* | 13 | 28491198 | rs2293941 | G | A | 0.417594 | 0.098711 | 2.33×10^-5^ | 0.458882 | 0 | 0.4629 |
| FG | *TOMM22P3* | 13 | 33554302 | rs576674 | G | A | -0.44406 | 0.147568 | 2.62×10^-3^ | 0.873439 | 0 | 0.5293 |
| FG | *WARS1* | 14 | 100839261 | rs3783347 | G | T | -0.18215 | 0.167075 | 2.76×10^-1^ | 0.096857 | 56.8 | 0.09863 |
| FG | *NPM1P47* | 15 | 62383155 | rs4502156 | T | C | -0.23047 | 0.098344 | 1.91×10^-2^ | 0.485988 | 27.9 | 0.2496 |
| FG | *DHRS7B* | 17 | 21073289 | rs118084662 | A | G | 0.121639 | 0.411434 | 7.68×10^-1^ | 0.012583 | 31.4 | 0.2327 |
| FG | *LNCNEF* | 20 | 22581268 | rs6048216 | T | C | -0.70521 | 0.125503 | 1.92×10^-8^ | 0.190365 | 0 | 0.4592 |
| FG | *DSCAM* | 21 | 41939569 | rs455489 | A | C | 0.000646 | 0.238584 | 9.98×10^-1^ | 0.042756 | 61.6 | 0.07385 |
| HbA1c | *TMEM79* | 1 | 156255456 | rs6684514 | G | A | -0.03594 | 0.002361 | 2.56×10^-52^ | 0.212549 | 0 | 0.7305 |
| HbA1c | *SPTA1* | 1 | 158626378 | rs857691 | C | T | 0.014333 | 0.002088 | 6.70×10^-12^ | 0.325222 | 82.2 | 0.003687 |
| HbA1c | *ATAD2B* | 2 | 24021231 | rs17509001 | T | C | 0.011995 | 0.008889 | 1.77×10^-1^ | 0.013197 | 50.8 | 0.1312 |
| HbA1c | *KRTCAP2P1, SIX3* | 2 | 45192080 | rs12712928 | G | C | 0.027315 | 0.001977 | 2.09×10^-43^ | 0.423123 | 0 | 0.6325 |
| HbA1c | *G6PC2* | 2 | 169763148 | rs560887 | T | C | 0.034282 | 0.006654 | 2.58×10^-7^ | 0.976991 | 34.1 | 0.2194 |
| HbA1c | *LINC00690* | 3 | 12267648 | rs7616006 | A | G | -0.00304 | 0.002144 | 1.56×10^-1^ | 0.291713 | 51.7 | 0.1264 |
| HbA1c | *SLC2A2* | 3 | 170724883 | rs8192675 | T | C | -0.01999 | 0.002405 | 9.46×10^-17^ | 0.204862 | 0 | 0.8509 |
| HbA1c | *IGF2BP2* | 3 | 185518921 | rs76922886 | G | A | 0.008939 | 0.002115 | 2.38×10^-5^ | 0.305273 | 0 | 0.5245 |
| HbA1c | *USP4* | 3 | 49382925 | rs9818758 | G | A | -0.00242 | 0.004 | 5.46×10^-1^ | 0.060026 | 0 | 0.9367 |
| HbA1c | *FREM3* | 4 | 144659795 | rs13134327 | G | A | 0.004479 | 0.002213 | 4.30×10^-2^ | 0.263115 | 0 | 0.9474 |
| HbA1c | *CDKAL1* | 6 | 20675792 | rs35261542 | C | A | 0.023255 | 0.001991 | 1.65×10^-31^ | 0.413167 | 0 | 0.3826 |
| HbA1c | *CCDC162P* | 6 | 109562035 | rs11964178 | A | G | -0.00537 | 0.00228 | 1.86×10^-2^ | 0.245248 | 0 | 0.5134 |
| HbA1c | *HBS1L* | 6 | 135418916 | rs7776054 | A | G | -0.02741 | 0.002067 | 3.95×10^-40^ | 0.340758 | 82.4 | 0.003439 |
| HbA1c | *MIR129-1* | 7 | 127841626 | rs4728092 | C | A | 0.012418 | 0.003194 | 1.01×10^-4^ | 0.112819 | 0 | 0.4983 |
| HbA1c | *YKT6* | 7 | 44235668 | rs4607517 | G | A | 0.02936 | 0.002372 | 3.44×10^-35^ | 0.210306 | 0 | 0.5928 |
| HbA1c | *ANK1* | 8 | 41630405 | rs4737009 | G | A | 0.019487 | 0.001956 | 2.19×10^-23^ | 0.440431 | 80 | 0.006752 |
| HbA1c | *SLC30A8* | 8 | 118185733 | rs11558471 | A | G | -0.01969 | 0.001957 | 7.95×10^-24^ | 0.447656 | 14.4 | 0.3109 |
| HbA1c | *SLC20A2* | 8 | 42383084 | rs6980507 | G | A | 0.011094 | 0.00197 | 1.78×10^-08^ | 0.569075 | 0 | 0.7228 |
| HbA1c | *CDKN2B-AS1* | 9 | 22130065 | rs10965243 | A | G | -0.01944 | 0.001987 | 1.30×10^-22^ | 0.418884 | 0 | 0.9913 |
| HbA1c | *ABO* | 9 | 136154168 | rs579459 | T | C | 0.009499 | 0.00251 | 1.54×10^-4^ | 0.267114 | 74.2 | 0.02059 |
| HbA1c | *INPP5E* | 9 | 139328722 | rs7851507 | G | T | 0.009806 | 0.002236 | 1.16×10^-5^ | 0.260796 | 90.2 | 3.72E-05 |
| HbA1c | *CDC123* | 10 | 12253597 | rs12221133 | G | A | -0.01062 | 0.002159 | 8.68×10^-7^ | 0.443710 | 96.5 | 3.85E-13 |
| HbA1c | *Y_RNA, HHEX* | 10 | 94466439 | rs12219514 | A | G | 0.007158 | 0.002097 | 6.43×10^-4^ | 0.870169 | 94.1 | 4.80E-08 |
| HbA1c | *TCF7L2* | 10 | 114754088 | rs7901695 | T | C | -0.0111 | 0.003217 | 5.58×10^-4^ | 0.046450 | 71.6 | 0.02973 |
| HbA1c | *HK1* | 10 | 71091013 | rs10823343 | A | G | 0.009879 | 0.003811 | 9.53×10^-3^ | 0.153003 | 76.6 | 0.01397 |
| HbA1c | *ARAP1* | 11 | 72432985 | rs11603334 | G | A | -0.00552 | 0.003014 | 6.69×10^-2^ | 0.038080 | 65.9 | 0.05318 |
| HbA1c | *MTNR1B* | 11 | 92708710 | rs10830963 | C | G | 0.009658 | 0.00347 | 5.38×10^-3^ | 0.421125 | 94.3 | 2.27E-08 |
| HbA1c | *KCNQ1* | 11 | 2858440 | rs2237896 | G | A | -0.02432 | 0.002064 | 4.76×10^-32^ | 0.403453 | 0 | 0.7717 |
| HbA1c | *FADS2* | 11 | 61619829 | rs174594 | C | A | 0.012661 | 0.001989 | 1.96×10^-10^ | 0.580290 | 57.3 | 0.09634 |
| HbA1c | *SINHCAF* | 12 | 31466613 | rs147538848 | G | A | 0.014802 | 0.002506 | 3.49×10^-9^ | 0.189345 | 0 | 0.5218 |
| HbA1c | *COL2A1, SENP1* | 12 | 48409054 | rs12819124 | C | A | -0.00923 | 0.003521 | 8.75×10^-3^ | 0.085340 | 38.7 | 0.1957 |
| HbA1c | *ATP11A* | 13 | 113351662 | rs282587 | G | A | -0.03186 | 0.008065 | 7.82×10^-5^ | 0.984398 | 0 | 0.955 |
| HbA1c | *HMG20A* | 15 | 77799657 | rs4886869 | A | G | 0.010824 | 0.002001 | 6.31×10^-8^ | 0.399140 | 69.2 | 0.03896 |
| HbA1c | *FAM234A* | 16 | 293562 | rs11248914 | T | C | -0.0089 | 0.001998 | 8.41×10^-6^ | 0.607096 | 0 | 0.9531 |
| HbA1c | *FTO* | 16 | 53803574 | rs1558902 | T | A | 0.005185 | 0.002452 | 3.44×10^-2^ | 0.195987 | 79.8 | 0.007156 |
| HbA1c | *PIEZO1* | 16 | 88853729 | rs837763 | C | T | 0.025298 | 0.001997 | 8.92×10^-37^ | 0.623320 | 82.8 | 0.002955 |
| HbA1c | *ERAL1* | 17 | 27183104 | rs9914988 | G | A | 0.012048 | 0.001961 | 8.12×10^-10^ | 0.551768 | 75.6 | 0.01657 |
| HbA1c | *HROB, ASB16* | 17 | 42241929 | rs12602486 | T | G | -0.01034 | 0.003096 | 8.38×10^-4^ | 0.111487 | 0 | 0.4665 |
| HbA1c | *TMC6* | 17 | 76121864 | rs2748427 | A | G | 0.04666 | 0.002577 | 2.99×10^-73^ | 0.188480 | 0 | 0.4692 |
| HbA1c | *FN3K* | 17 | 80694826 | rs8067360 | T | C | 0.02974 | 0.001937 | 3.49×10^-53^ | 0.505095 | 58.7 | 0.08883 |
| HbA1c | *MYO9B* | 19 | 17256523 | rs17533903 | G | A | 0.009543 | 0.003116 | 2.19×10^-3^ | 0.105150 | 84.6 | 0.001487 |
| HbA1c | *RN7SL836P* | 19 | 46159986 | rs57601949 | C | T | 0.007926 | 0.002111 | 1.74×10^-4^ | 0.431709 | 47.1 | 0.1512 |
| HbA1c | *TMPRSS6* | 22 | 37469591 | rs4820268 | G | A | -0.01299 | 0.001993 | 7.13×10^-11^ | 0.455693 | 0 | 0.7171 |
| FC | *LYPLAL1-AS1, ZC3H11B* | 1 | 2.2E+08 | rs4846565 | G | A | 0.009441 | 0.015885 | 5.52×10^-1^ | 0.363512 | - | - |
| FC | *MAP3K19* | 2 | 1.36E+08 | rs1530559 | A | G | 0.005295 | 0.035139 | 8.80×10^-1^ | 0.924597 | - | - |
| FC | *NEU2* | 2 | 2.27E+08 | rs2943645 | C | T | 0.038661 | 0.026888 | 1.51×10^-1^ | 0.908764 | - | - |
| FC | *GCKR* | 2 | 27741237 | rs780094 | T | C | 0.028882 | 0.015602 | 6.43×10^-2^ | 0.446279 | - | - |
| FC | *COBLL1* | 2 | 1.66E+08 | rs10195252 | T | C | -0.06471 | 0.029568 | 2.87×10^-2^ | 0.073341 | - | - |
| FC | *PPARG* | 3 | 12390484 | rs17036328 | T | C | -0.03554 | 0.046924 | 4.49×10^-1^ | 0.027011 | - | - |
| FC | *TET2* | 4 | 1.06E+08 | rs974801 | A | G | 0.030388 | 0.015842 | 5.53×10^-2^ | 0.62455 | - | - |
| FC | *FAM13A* | 4 | 89741269 | rs3822072 | G | A | 0.003089 | 0.01582 | 8.45×10^-1^ | 0.633965 | - | - |
| FC | *C5orf67, RPL26P19* | 5 | 55806751 | rs459193 | A | G | 0.020484 | 0.015325 | 1.82×10^-1^ | 0.466907 | - | - |
| FC | *UHRF1BP1* | 6 | 34764922 | rs6912327 | T | C | -0.00127 | 0.015948 | 9.36×10^-1^ | 0.363614 | - | - |
| FC | *RSPO3* | 6 | 1.27E+08 | rs2745353 | C | T | 0.033626 | 0.01528 | 2.79×10^-2^ | 0.570554 | - | - |
| FC | *HIP1* | 7 | 75176196 | rs1167800 | G | A | -0.02081 | 0.028187 | 4.60×10^-1^ | 0.678352 | - | - |
| FC | *RNU6-526P, RNU6-1151P* | 8 | 9183596 | rs4841132 | A | G | 0.07969 | 0.059278 | 1.79×10^-1^ | 0.952374 | - | - |
| FC | *TCF7L2* | 10 | 1.15E+08 | rs7903146 | C | T | -0.00467 | 0.034416 | 8.92×10^-1^ | 0.05102 | - | - |
| FC | *IGF1* | 12 | 1.03E+08 | rs35747 | G | A | 0.020081 | 0.016332 | 2.19×10^-1^ | 0.670768 | - | - |
| FC | *FTO* | 16 | 53800954 | rs1421085 | T | C | 0.043691 | 0.019219 | 2.31×10^-2^ | 0.204082 | - | - |
| FC | *PEPD* | 19 | 33899065 | rs731839 | G | A | -0.0348 | 0.014871 | 1.94×10^-2^ | 0.471489 | - | - |

Abbreviations: ALT, alternative allele; Chr, chromosome; EAF, effect allele frequency; FC, fasting C-peptide; FG, fasting glucose; HbA1c, haemoglobin A1c; REF, reference allele;

SE, standard error.

^†^P value for heterogeneity.

**Supplementary Table S3. Single nucleotide polymorphisms used as instrumental variables for colorectal cancer**

| **Trait** | **Chr** | **Chr position (hg19)** | **rsID** | **REF** | **ALT** | **Effect** | **SE** | **P-value** | **I^2^** | **q-pvalue**^†^ |
| --- | --- | --- | --- | --- | --- | --- | --- | --- | --- | --- |
| FG | 1 | 214159256 | rs340874 | T | C | -0.00428 | 0.01843 | 0.816322 | 0 | 0.600193 |
| FG | 2 | 45188353 | rs895636 | C | T | -0.01152 | 0.018775 | 0.539626 | 0 | 0.776672 |
| FG | 2 | 27741237 | rs780094 | T | C | 0.025502 | 0.018397 | 0.165693 | 0 | 0.424805 |
| FG | 2 | 169763148 | rs560887 | T | C | -0.09301 | 0.058133 | 0.109608 | 8.78336 | 0.359984 |
| FG | 2 | 27152874 | rs1371614 | C | T | -0.00168 | 0.024889 | 0.946252 | 18.4811 | 0.293441 |
| FG | 3 | 170713290 | rs1280 | T | C | 0.038543 | 0.062624 | 0.538247 | 0 | 0.434326 |
| FG | 3 | 185513392 | rs7651090 | A | G | -0.0228 | 0.019409 | 0.240114 | 10.0157 | 0.351788 |
| FG | 5 | 76425867 | rs7708285 | G | A | 0.031037 | 0.067083 | 0.643609 | 0 | 0.422771 |
| FG | 5 | 95539448 | rs4869272 | C | T | 0.023 | 0.021154 | 0.276914 | 0 | 0.658501 |
| FG | 6 | 20685486 | rs9356744 | T | C | -0.023 | 0.018404 | 0.211494 | 14.4536 | 0.321617 |
| FG | 6 | 7213200 | rs17762454 | C | T | 0.029916 | 0.018434 | 0.104613 | 0 | 0.625085 |
| FG | 7 | 15064309 | rs2191349 | G | T | -0.01369 | 0.01965 | 0.486066 | 48.3845 | 0.084605 |
| FG | 7 | 44245853 | rs917793 | A | T | 0.009572 | 0.022137 | 0.665446 | 45.7878 | 0.100493 |
| FG | 8 | 40484239 | rs2722425 | T | C | -0.00849 | 0.020271 | 0.675496 | 0 | 0.584561 |
| FG | 8 | 9177732 | rs983309 | T | G | - | - | - | - | - |
| FG | 8 | 118185733 | rs11558471 | A | G | -0.0368 | 0.018401 | 0.0455 | 41.5345 | 0.128322 |
| FG | 9 | 22134094 | rs10811661 | T | C | -0.00627 | 0.019957 | 0.753485 | 6.99119 | 0.371753 |
| FG | 9 | 4293150 | rs10814916 | A | C | 0.00978 | 0.018693 | 0.600862 | 0 | 0.992868 |
| FG | 9 | 622523 | rs10815355 | G | T | 0.000603 | 0.028136 | 0.982895 | 0 | 0.825997 |
| FG | 9 | 139256766 | rs3829109 | G | A | 0.006794 | 0.027492 | 0.804814 | 16.6186 | 0.306556 |
| FG | 10 | 113042093 | rs10885122 | T | G | -0.01432 | 0.030308 | 0.636473 | 0 | 0.441275 |
| FG | 10 | 114754088 | rs7901695 | T | C | -0.05236 | 0.044621 | 0.240594 | 0 | 0.615429 |
| FG | 11 | 61571478 | rs174550 | T | C | -0.10741 | 0.018426 | 5.57×10^-9^ | 0 | 0.944032 |
| FG | 11 | 72432985 | rs11603334 | G | A | -0.03304 | 0.046086 | 0.473446 | 0 | 0.618419 |
| FG | 11 | 92708710 | rs10830963 | C | G | 0.001348 | 0.018511 | 0.941943 | 0.737942 | 0.411361 |
| FG | 11 | 47346723 | rs11039182 | T | C | 0.123338 | 0.079736 | 0.121902 | 5.56831 | 0.380968 |
| FG | 12 | 56865338 | rs2657879 | A | G | 0.057787 | 0.028537 | 0.042866 | 0 | 0.43786 |
| FG | 13 | 28491198 | rs2293941 | G | A | 0.015434 | 0.018374 | 0.400916 | 0 | 0.909926 |
| FG | 13 | 33554302 | rs576674 | G | A | 0.047396 | 0.027389 | 0.083553 | 13.971 | 0.324945 |
| FG | 14 | 100839261 | rs3783347 | G | T | 0.025668 | 0.030137 | 0.394366 | 11.8108 | 0.339703 |
| FG | 15 | 62383155 | rs4502156 | T | C | -0.03122 | 0.018354 | 0.088964 | 11.2537 | 0.343472 |
| FG | 17 | 21073289 | rs118084662 | A | G | - | - | - | - | - |
| FG | 20 | 22581268 | rs6048216 | T | C | 0.009447 | 0.022903 | 0.679991 | 0 | 0.61513 |
| FG | 21 | 41939569 | rs455489 | A | C | 0.021201 | 0.044068 | 0.630442 | 0 | 0.7578 |
| HbA1c | 1 | 156255456 | rs6684514 | G | A | 0.010283 | 0.022108 | 0.641864 | 2.47173 | 0.400612 |
| HbA1c | 1 | 158626378 | rs857691 | C | T | 0.005558 | 0.019333 | 0.773758 | 0 | 0.7936 |
| HbA1c | 2 | 24021231 | rs17509001 | T | C | 0.050103 | 0.074382 | 0.500576 | 10.5202 | 0.348409 |
| HbA1c | 2 | 45192080 | rs12712928 | G | C | -0.00958 | 0.018812 | 0.610665 | 0 | 0.808799 |
| HbA1c | 2 | 169763148 | rs560887 | T | C | -0.09301 | 0.058133 | 0.109608 | 8.78336 | 0.359984 |
| HbA1c | 3 | 12267648 | rs7616006 | A | G | 0.002904 | 0.020238 | 0.885885 | 0 | 0.634422 |
| HbA1c | 3 | 170724883 | rs8192675 | T | C | 0.035569 | 0.022138 | 0.108122 | 0 | 0.674978 |
| HbA1c | 3 | 185518921 | rs76922886 | G | A | -0.02297 | 0.019412 | 0.236654 | 10.274 | 0.350059 |
| HbA1c | 3 | 49382925 | rs9818758 | G | A | 0.051194 | 0.039271 | 0.192367 | 0 | 0.906397 |
| HbA1c | 4 | 144659795 | rs13134327 | G | A | 0.020351 | 0.021077 | 0.334252 | 0 | 0.46226 |
| HbA1c | 6 | 20675792 | rs35261542 | C | A | -0.02452 | 0.019184 | 0.201282 | 13.411 | 0.328793 |
| HbA1c | 6 | 109562035 | rs11964178 | A | G | -0.01169 | 0.021224 | 0.58162 | 16.5468 | 0.307058 |
| HbA1c | 6 | 135418916 | rs7776054 | A | G | 0.061013 | 0.01927 | 0.001545 | 0 | 0.602 |
| HbA1c | 7 | 127841626 | rs4728092 | C | A | 0.009621 | 0.030988 | 0.756197 | 73.7328 | 0.001893 |
| HbA1c | 7 | 44235668 | rs4607517 | G | A | 0.006496 | 0.022049 | 0.768287 | 41.6672 | 0.127425 |
| HbA1c | 8 | 41630405 | rs4737009 | G | A | -0.02128 | 0.022141 | 0.336487 | 29.1017 | 0.216775 |
| HbA1c | 8 | 118185733 | rs11558471 | A | G | -0.0368 | 0.018401 | 0.0455 | 41.5345 | 0.128322 |
| HbA1c | 8 | 42383084 | rs6980507 | G | A | -0.00578 | 0.018508 | 0.754761 | 15.0161 | 0.317724 |
| HbA1c | 9 | 22130065 | rs10965243 | A | G | -0.01728 | 0.019258 | 0.369616 | 42.3737 | 0.12268 |
| HbA1c | 9 | 136154168 | rs579459 | T | C | -0.01517 | 0.020303 | 0.455025 | 0 | 0.863818 |
| HbA1c | 9 | 139328722 | rs7851507 | G | T | -0.00331 | 0.02127 | 0.876332 | 21.1091 | 0.274717 |
| HbA1c | 10 | 12253597 | rs12221133 | G | A | -0.01938 | 0.018425 | 0.292978 | 63.4862 | 0.017679 |
| HbA1c | 10 | 94466439 | rs12219514 | A | G | 0.007331 | 0.026641 | 0.783195 | 0 | 0.779281 |
| HbA1c | 10 | 114754088 | rs7901695 | T | C | -0.05236 | 0.044621 | 0.240594 | 0 | 0.615429 |
| HbA1c | 10 | 71091013 | rs10823343 | A | G | 0.027744 | 0.02504 | 0.267877 | 0 | 0.49169 |
| HbA1c | 11 | 72432985 | rs11603334 | G | A | -0.03304 | 0.046086 | 0.473446 | 0 | 0.618419 |
| HbA1c | 11 | 92708710 | rs10830963 | C | G | 0.001348 | 0.018511 | 0.941943 | 0.737942 | 0.411361 |
| HbA1c | 11 | 2858440 | rs2237896 | G | A | 0.007605 | 0.018434 | 0.679919 | 0 | 0.631633 |
| HbA1c | 11 | 61619829 | rs174594 | C | A | 0.11097 | 0.019221 | 7.76E-09 | 0 | 0.877069 |
| HbA1c | 12 | 31466613 | rs147538848 | G | A | -0.0325 | 0.024722 | 0.18867 | 48.7498 | 0.08245 |
| HbA1c | 12 | 48409054 | rs12819124 | C | A | -0.03116 | 0.032263 | 0.334161 | 0 | 0.964923 |
| HbA1c | 13 | 113351662 | rs282587 | G | A | 0.063939 | 0.071193 | 0.369133 | 6.40409 | 0.375569 |
| HbA1c | 15 | 77799657 | rs4886869 | A | G | -0.01194 | 0.018425 | 0.517052 | 0 | 0.568774 |
| HbA1c | 16 | 293562 | rs11248914 | T | C | -0.01019 | 0.01841 | 0.579862 | 0 | 0.884572 |
| HbA1c | 16 | 53803574 | rs1558902 | T | A | 0.05557 | 0.022903 | 0.015254 | 0 | 0.576636 |
| HbA1c | 16 | 88853729 | rs837763 | C | T | 0.017692 | 0.018473 | 0.338217 | 0 | 0.579403 |
| HbA1c | 17 | 27183104 | rs9914988 | G | A | 0.027494 | 0.018427 | 0.135671 | 0.688996 | 0.411661 |
| HbA1c | 17 | 42241929 | rs12602486 | T | G | -0.04415 | 0.029316 | 0.132089 | 0 | 0.820124 |
| HbA1c | 17 | 76121864 | rs2748427 | A | G | 0.060139 | 0.027007 | 0.025962 | 0 | 0.50104 |
| HbA1c | 17 | 80694826 | rs8067360 | T | C | -0.01771 | 0.018376 | 0.335207 | 0 | 0.772246 |
| HbA1c | 19 | 17256523 | rs17533903 | G | A | -0.00689 | 0.031308 | 0.825775 | 51.3893 | 0.06753 |
| HbA1c | 19 | 46159986 | rs57601949 | C | T | -0.02413 | 0.018592 | 0.194332 | 0 | 0.839558 |
| HbA1c | 22 | 37469591 | rs4820268 | G | A | 0.013887 | 0.018378 | 0.449874 | 47.0674 | 0.092545 |
| FC | 1 | 2.2E+08 | rs4846565 | G | A | -0.00594 | 0.018502 | 0.74803 | 0 | 0.452903 |
| FC | 2 | 1.36E+08 | rs1530559 | A | G | 0.009937 | 0.046844 | 0.83201 | 0 | 0.551121 |
| FC | 2 | 2.27E+08 | rs2943645 | C | T | 0.04643 | 0.033263 | 0.162754 | 0 | 0.540561 |
| FC | 2 | 27741237 | rs780094 | T | C | 0.025502 | 0.018397 | 0.165693 | 0 | 0.424805 |
| FC | 2 | 1.66E+08 | rs10195252 | T | C | 0.006149 | 0.033204 | 0.853073 | 0 | 0.819185 |
| FC | 3 | 12390484 | rs17036328 | T | C | 0.066816 | 0.051932 | 0.19823 | 34.847 | 0.175126 |
| FC | 4 | 1.06E+08 | rs974801 | A | G | 0.008012 | 0.018425 | 0.663671 | 0 | 0.442235 |
| FC | 4 | 89741269 | rs3822072 | G | A | -0.00559 | 0.019267 | 0.771839 | 46.9039 | 0.093547 |
| FC | 5 | 55806751 | rs459193 | A | G | 0.035203 | 0.018359 | 0.05518 | 0.417322 | 0.413328 |
| FC | 6 | 34764922 | rs6912327 | T | C | -0.02583 | 0.019342 | 0.181704 | 60.816 | 0.025732 |
| FC | 6 | 1.27E+08 | rs2745353 | C | T | 0.000683 | 0.018382 | 0.970349 | 0 | 0.483207 |
| FC | 7 | 75176196 | rs1167800 | G | A | -0.01279 | 0.019791 | 0.518119 | 0 | 0.971937 |
| FC | 8 | 9183596 | rs4841132 | A | G | - | - | - | - | - |
| FC | 10 | 1.15E+08 | rs7903146 | C | T | -0.05725 | 0.044646 | 0.199706 | 0 | 0.695057 |
| FC | 12 | 1.03E+08 | rs35747 | G | A | -0.02696 | 0.019331 | 0.163035 | 0 | 0.857074 |
| FC | 16 | 53800954 | rs1421085 | T | C | 0.053526 | 0.022898 | 0.019411 | 0 | 0.571526 |
| FC | 19 | 33899065 | rs731839 | G | A | -0.00526 | 0.018349 | 0.774469 | 0 | 0.738187 |

Abbreviations: ALT, alternative allele; Chr, chromosome; EAF, effect allele frequency; FC, fasting C-peptide; FG, fasting glucose; HbA1c, haemoglobin A1c; REF, reference allele;

SE, standard error.

^†^P value for heterogeneity.

**Supplementary Table S4. Study description in terms of exposure (fasting glucose, HbA1c, and fasting C-peptide)**

| **Study** | **Description** |
| --- | --- |
| JPHC | The JPHC Study was initiated in 1990 (cohort I) and 1993–1994 (cohort II). All subjects were of Japanese descent. They were recruited from 11 public health centres. At the time of their first (baseline) survey, the subjects were aged 40–59 years in 1990 (cohort I) and 40–69 years in 1993 (cohort II). The JPHC Study has been described in detail previously [PMID: 25104790]. In the current study, the subjects were drawn from a cohort of 33,736 residents in 9 public health centres. These individuals not only returned the self-administered questionnaires but also donated 10 mL of venous blood at the time of the baseline survey. In the first step of sample selection, we stratified the cohort according to the sex of the subjects, the 5-year age categories, and the 9 public health centres. We performed random sampling by selecting a similar proportion of subjects from each stratum. Subsequently, we identified 12,645 subjects for inclusion in genotyping, following which we performed the standard quality control of GWAS (see Table S3). Prior to the performance of genetic research on the JPHC samples, we obtained approval from the institutional review board of the National Cancer Center (Approval No.: 2011-044), Tokyo, Japan, and provided eligible subjects with the option of refusing participation in the research. |
| TMM | The Tohoku Medical Megabank Community-Based Cohort (TMM CommCohort) study was designed as previously described [PMID: 31932529]. Briefly, 20–75-year-old residents from Iwate and Miyagi, Pacific coast prefectures in Northeast Japan, were recruited between May 2013 and March 2016. The customised genotyping array designed by the TMM based on the Axiom platform (Thermo Fisher Scientific, Waltham, MA USA), denoted as Japonica array version 2 (JPAv2), was used for the genotyping of the 53,599 participants. After quality control, 40,797 subjects remained and were considered for subsequent analysis. Genotype imputation was performed for 5,000 subjects and the remaining subjects were included in the last group. The imputed datasets were combined by qctool v2.0.8, and 81,706,044 variants were finally considered for GWAS. The subjects who lacked phenotypes or covariates were removed before each analysis. The TMM CommCohort Study was approved by the Institutional Review Boards of the Iwate Medical University and Tohoku University, and all participants provided written informed consent. The study was conducted in accordance with the Declaration of Helsinki. |
| J-MICC | In the J-MICC Study [PMID: 32963210], 14,550 eligible participants were selected for GWAS from 13 study areas throughout Japan, considering an area distribution of respondents and minimum sample size (n = 500) for one area. The participants filled in a questionnaire and donated blood samples for genotyping and accounted for 15.7% of the 92,527 participants in the survey. A total of 14,531 DNA samples were successfully genotyped for GWAS. The standard quality control for GWAS was further performed. The J-MICC study, including genotyping, was approved by the ethics committee of the Nagoya University Graduate School of Medicine (Approval No.: 2010-0939), Nagoya, Japan, and written informed consent was obtained from all the participants in the present study. |

Abbreviations: HbA1c, haemoglobin A1c; GWAS, genome-wide association study; J-MICC, the Japan Multi-Institutional Collaborative Cohort; JPHC, Japan Public Health Center; TMM, the Tohoku Medical Megabank.

**Supplementary Table S5. Information on genotyping methods, quality controls for single nucleotide polymorphisms, imputation, and statistical analysis for studies**

|  | **Study name** | **Genotyping** | | | | | **Imputation** | | **Statistical analysis** | |
| --- | --- | --- | --- | --- | --- | --- | --- | --- | --- | --- |
|  |  | **Platform** | **QC** | | | | **Software** | **Reference** | **Software** | **Covariates** |
|  |  |  | **MAF** | **Call rate** | **HWE** | **Other criteria** |  |  |  |  |
| Exposure | JPHC | Illumina HumanOmni2.5  /Express/ExpressExome | ≥1% | ≥98% | ≥1×10^-6^ | Sex mismatches, related samples  (IBD≥0.1875) | SHAPEIT2  IMPUTE2 | 1000G phase3v5;  individuals of ALL | SNP  TEST | age, sex,  top5PC |
|  | TMM | Axiom Japonica array version 2 (JPAv2) | ≥1% | ≥99% | ≥1×10^-4^ | Sex mismatches, related samples  (IBD≥0.1875) | SHAPEIT2  IMPUTE2 | 1000 Genomes phase3v5;  individuals of ALL | SNP  TEST | age, sex,  top5PC |
|  | J-MICC | Illumina HumanOmni  ExpressExome | ≥1% | ≥98% | ≥1×10^-6^ | Sex mismatches, related samples  (IBD≥0.1875) | SHAPEIT2  IMPUTE2 | 1000G phase3v5;  individuals of ALL | SNP  TEST | age, sex,  top5PC |
| Outcome | JPHC-base | Illumina HumanOmni2.5  /Express/ExpressExome | ≥1% | ≥98% | ≥1×10^-6^ | Sex mismatches, related samples  (IBD≥0.1875) | SHAPEIT2  IMPUTE2 | 1000G phase3v5;  individuals of ALL | SNP  TEST | age, sex,  top10PC |
|  | JPHC-5 year | Illumina Human  OmniExpressExome | ≥1% | ≥98% | ≥1×10^-6^ |  | SHAPEIT2  IMPUTE2 | 1000G phase3v5;  individuals of ALL | SNP  TEST | age, sex,  top10PC |
|  | NAGANO | Illumina1M-duo | ≥1% | ≥98% | ≥1×10^-6^ | Sex mismatches, related samples  (IBD≥0.1875) | SHAPEIT2  IMPUTE2 | 1000G phase3v5;  individuals of ALL | SNP  TEST | age, sex,  top3PC |
|  | HERPACC | Illumina Human CoreExome 12/24 | ≥1% | ≥98% | ≥1×10^-6^ | Sex mismatches, related samples  (IBD≥0.1875) | SHAPEIT2  minimac3 | 1000G phase3v5;  individuals of ALL | PLINK2 | age, sex,  top10PC |
|  | J-MICC | Illumina Asian Screening Array | ≥1% | ≥98% | ≥1×10^-6^ | Sex mismatches, related samples  (IBD≥0.1875) | SHAPEIT2  minimac3 | 1000G phase3v5;  individuals of ALL | SNP  TEST | age, sex,  top10PC |
|  | BBJ | Illumina OmniExpressExome  /OmniExpress/HumanExome | ≥1% | ≥99% | ≥1×10^-6^ | ND | MaCH  minimac3 | 1000G phase1;  individuals of JPT/CHS/CHD | ND | top2PC |

Abbreviations: BBJ, Biobank Japan; GWAS, genome-wide association study; HERPACC, Hospital-based Epidemiologic Research Program at Aichi Cancer Center; J-MICC, the Japan Multi-Institutional Collaborative Cohort; JPHC, Japan Public Health Center; PC, principal component; TMM, the Tohoku Medical Megabank.**Supplementary Table S6. Study description in terms of outcome (colorectal cancer)**

| **Study** | **Description** |
| --- | --- |
| JPHC-base | The JPHC Study was initiated in 1990 (cohort I) and 1993–1994 (cohort II). All subjects were of Japanese descent. They were recruited from 11 public health centres. At the time of their first (baseline) survey, the subjects were aged 40–59 years in 1990 (cohort I) and 40–69 years in 1993 (cohort II). The JPHC Study has been described in detail previously [PMID: 25104790]. A case-cohort design (called JPHC-base in this study) was applied to examine the association between glycaemic trait-associated SNPs and colorectal cancer risk. In this case-cohort study, 33,736 subjects from nine public health centres across Japan, who responded to the baseline questionnaire and provided blood samples during the health check-up, were recruited. Following the application of standard sample exclusion criteria and standard quality control for GWAS (see Table S3), 2,510 sub-cohort subjects (including 76 incident colorectal cancer cases) were chosen. Additionally, 406 incident colorectal cancer cases occurring outside the sub-cohort were included in the analysis. Among these 2,434 non-cases and 482 cases, we conducted an ordinary prospective logistic regression analysis to estimate the odds ratio and standard error adjusted for age, sex, and top 3 PCs. We used the ICD-O-3 code: C18,19,20, to identify the colorectal cancer cases. Our study was approved by the institutional review board of the National Cancer Center, Japan, and we provided eligible subjects with the option of refusing participation in the research. |
| JPHC-5year | The JPHC Study was initiated in 1990 (cohort I) and 1993–1994 (cohort II). All subjects were of Japanese descent. The subjects were recruited from 11 public health centres. At the time of their first (baseline) survey, the subjects were aged 40–59 years in 1990 (cohort I) and 40–69 years in 1993 (cohort II). The JPHC Study has been described in detail previously [PMID: 25104790]. A case-cohort design (called JPHC-5year in this study) was used to examine the association between glycaemic trait- associated SNPs and colorectal cancer risk. In this case-cohort study, 10,950 subjects who responded to the five-year-follow up questionnaire and provided blood samples during the health check-up were recruited from nine public health centres across Japan. This population did not take part in the baseline questionnaire, and hence these participants were not included in JPHC-base. Following the application of standard sample exclusion criteria and standard quality control for GWAS (see Table 3), 3,688 sub-cohort subjects (including 81 incident cancer cases) were randomly chosen and 113 incident colorectal cancer cases occurring outside of the sub-cohort were included in the analysis. Among these, 3607 subjects were non-cases and 194 subjects were cases. We conducted an ordinary prospective logistic regression analysis to estimate the odds ratio and standard error adjusted for age, sex, and top 3 PCs. We used the ICD-O-3 code: C18,19,20, to identify the colorectal cancer cases. The study was approved by the institutional review board of the National Cancer Center, Japan, and provided eligible subjects with the option of refusing participation in the research. |
| NAGANO | A hospital-based case-control study of gastrointestinal cancer (called NAGANO Study) was conducted between October 1998 and March 2002, in four hospitals, in Nagano Prefecture, Japan [PMID: 4690789]. The eligible cases included colorectal cancer patients aged 20–74 years newly diagnosed during the survey in the hospitals. We recruited 121 colorectal cancer patients to the study; none of the patients refused to participate in the study. We selected controls from the medical check-up examinees in the four hospitals. Eligible healthy controls were those confirmed with no cancer during the medical check-up, which included upper gastrointestinal endoscopy or X-ray, faecal occult blood test, and abdominal ultrasound. Following screening during the medical check-up, the subjects were confirmed using a detailed check-up. The controls were chosen from the respective hospitals and matched for sex, age (within 3 years), and residential area during the study period. Following the application of standard sample exclusion criteria and standard quality control for GWAS (see Table 3), we identified 103 non-cases and 105 cases. We conducted logistic regression analysis to estimate the odds ratios and standard error adjusted for age, sex, and top 3 PCs. All cases were histopathologically confirmed according to the General Rules for Clinical and Pathological Studies on Cancer of the Colon, Rectum, and Anus. We obtained written informed consent from all cases and controls. Our study was approved by the Institutional Review Board of the National Cancer Center, Tokyo, Japan. |
| HERPACC | The HERPACC-2 study was conducted between January 2001 and November 2005 at Aichi Cancer Center. Details of HERPACC is described elsewhere [PMID: 12718640]. Briefly, all first-visit outpatients (n = 29,736) during the study period were asked to complete a self-administered questionnaire and provide blood samples. Of these, 28,776 (96.7%) participated in the study following written informed consent and 13,824 subjects (48.0% of participants) further provided blood samples. All the subjects were asked to fill out a self-administered questionnaire. Among 13,824 subjects, 7,053 were confirmed to have no detectable cancer and no history of neoplasia within a one-year window period from participation. We confirmed 553 cases of colorectal cancer incidence. The study protocol was approved by ethics committees at Aichi Cancer Center, Nagoya, Japan. For replication analysis for colorectal cancer MR analysis, genotyping data of randomly selected 163 colorectal cancer cases and 3,819 non-cancer subjects based on Illumina Human Core Exome 12/24 chips were used. |
| J-MICC | In the J-MICC Study [PMID: 32963210], participants were followed for cancer incidence from the baseline survey through 2015. Cancer cases were ascertained by using population- and/or hospital-based cancer registries, surveys in principal hospitals in study areas, and self-reports by participants followed by confirmation with medical records. Colorectal cancer was defined by the ICD-O-3 code C18, C19, and C20. Genotyping with Illumina Asian Screening Array was conducted in cancer cases and selected non-cancer participants. After excluding those with history of cancer at baseline, incidence of cancer other than colorectal cancer, incidence of cancer after 2015, genotype data based on the Illumina HumanOmni ExpressExome, and the data that failed to pass the standard quality control for GWAS, 306 cases of colorectal cancer and 921 non-cancer controls were included in the analysis of SNP-outcome associations. The J-MICC study, including genotyping, was approved by the ethics committee of the Nagoya University Graduate School of Medicine (Approval No.: 2010-0939), Nagoya, Japan, and written informed consent was obtained from all the participants in the present study. |
| BBJ | We used public summary-data in the BBJ study from JENGER (http://jenger.riken.jp/result). The study description is shown previously [PMID: 29471430]. |

Abbreviations: BBJ, Biobank Japan; GWAS, genome-wide association study; HERPACC, Hospital-based Epidemiologic Research Program at Aichi Cancer Center; J-MICC, the Japan Multi-Institutional Collaborative Cohort; JPHC, Japan Public Health Center; PC, principal component; TMM, the Tohoku Medical Megabank.

**Supplementary Table S7. Characteristics of the studies considered for the analysis of SNP-glycaemic trait associations**

| **Phenotype** | **Source** | **N** | **Age**  **mean ± SD** | **Women**  **%** |
| --- | --- | --- | --- | --- |
| **Fasting glucose levels** | JPHC | 3,537 | 54.0 ± 7.9 | 64.1 |
|  | TMM | 9,900 | 58.7 ± 11.7 | 66.4 |
|  | J-MICC | 3,852 | 53.3 ± 9.2 | 46.3 |
| **HbA1c** | JPHC | 8,207 | 63.0 ± 7.1 | 69.8 |
|  | TMM | 36,647 | 60.2 ± 11.2 | 63.4 |
|  | J-MICC | 7,948 | 55.5 ± 9.1 | 46.1 |
| **Fasting C-peptide levels** | JPHC | 1,666 | 54.5 ± 7.7 | 62.2 |

Abbreviations: J-MICC, the Japan Multi-Institutional Collaborative Cohort; JPHC, Japan Public Health Center; SD, standard deviation; SNP, single nucleotide polymorphism; TMM, the Tohoku Medical Megabank.**Supplementary Table S8. Characteristics of the studies considered for the analysis of SNP-colorectal cancer associations**

|  | **All** | | | **Cases** | | | **Controls** | | |
| --- | --- | --- | --- | --- | --- | --- | --- | --- | --- |
| **Source** | **N** | **Age**  **mean ± SD** | **Women**  **%** | **N** | **Age**  **mean ± SD** | **Women**  **%** | **N** | **Age**  **mean ± SD** | **Women**  **%** |
| JPHC-base | 2,916 | 54.3 ± 7.9 | 62.3 | 482 | 57.0 ± 7.3 | 49.0 | 2,434 | 53.8 ± 7.9 | 65.0 |
| JPHC-5year | 3,801 | 52.9 ± 7.4 | 57.8 | 194 | 55.5 ± 6.8 | 42.3 | 3,607 | 52.8 ± 7.4 | 58.6 |
| NAGANO | 208 | 59.3 ± 8.9 | 37.0 | 105 | 59.4 ± 9.0 | 37.1 | 103 | 59.2 ± 8.8 | 36.9 |
| HERPACC | 3,982 | 52.1 ± 12.2 | 50.1 | 163 | 59.4 ± 10.1 | 36.8 | 3,819 | 52.1 ± 12.2 | 50.5 |
| J-MICC | 1,201 | 56.6 ±9.5 | 47.5 | 300 | 60.0 ±6.5 | 38.0 | 901 | 55.4 ±10.0 | 50.6 |
| BBJ | 33,870 | - | 55.9 | 6,692 | 66.9 ± 10.1 | 36.3 | 27,178 | 60.7 ± 10.0 | 60.7 |

Abbreviations: BBJ, Biobank Japan; HERPACC, Hospital-based Epidemiologic Research Program at Aichi Cancer Center; J-MICC, the Japan Multi-Institutional Collaborative Cohort; JPHC, Japan Public Health Center; SD, standard deviation; SNP, single nucleotide polymorphism; TMM, the Tohoku Medical Megabank.

**Supplementary Figure S1. Flow chart of single nucleotide polymorphism selection from the literature**


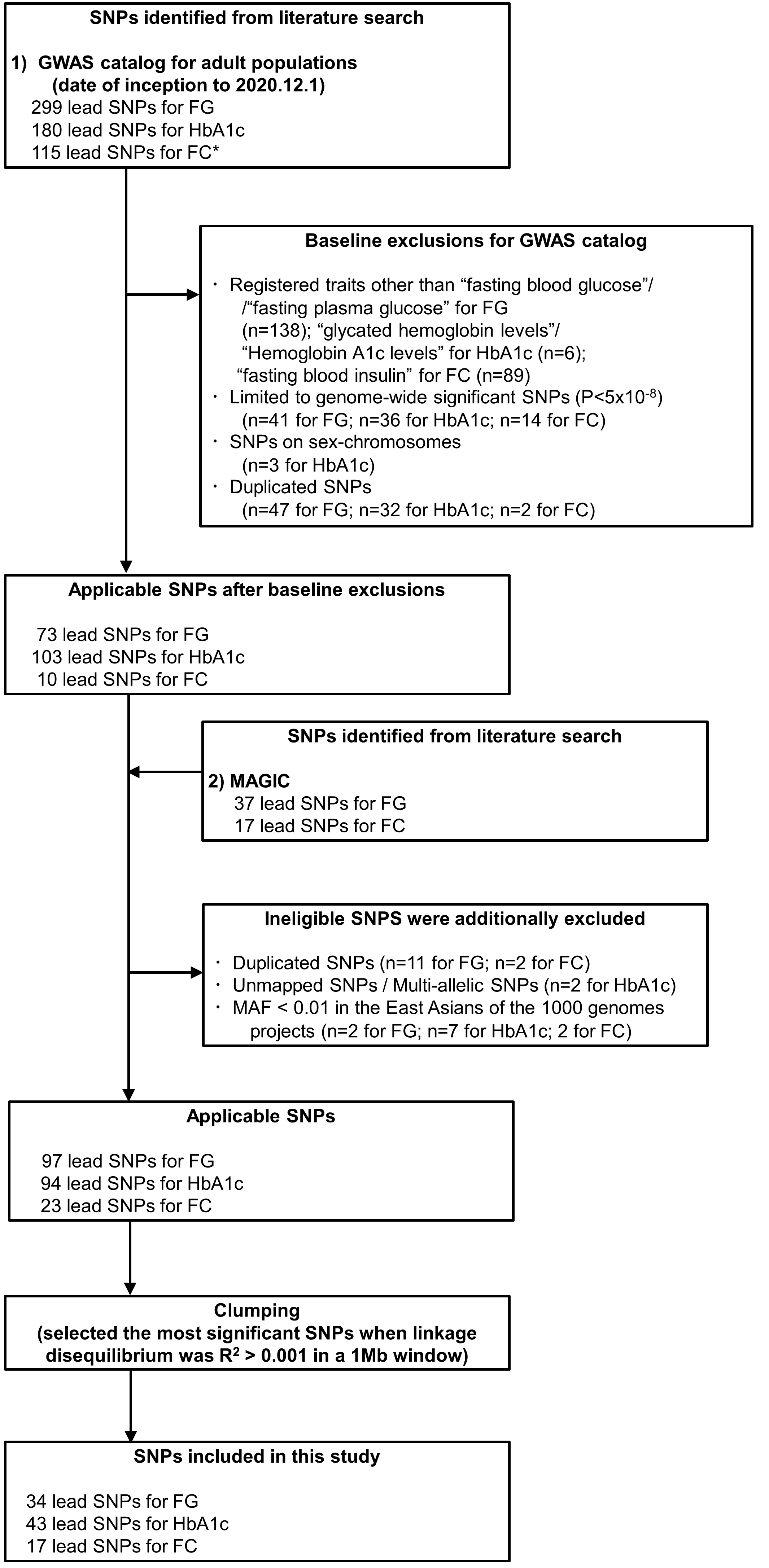


*Due to the unavailability of selecting SNPs for FC, we substituted fasting insulin for FC when selecting instrumental variables.

Abbreviation: FC, fasting C-peptide; FG, fasting glucose; HbA1c, haemoglobin A1c; MAGIC, the Meta-Analysis of Glucose and Insulin related traits Consortium; SNP, single nucleotide polymorphism.

**Supplementary Figure S2. Analyses of fasting glucose and colorectal cancer risk**

a). Scatter plots of genetic association with colorectal cancer (outcome) over genetic association with fasting glucose (exposure).


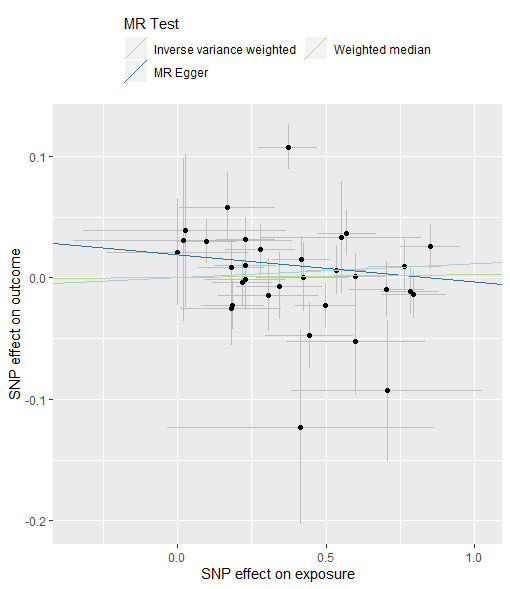


Abbreviation: SNP, single nucleotide polymorphism.

b). Leave-one-out sensitivity analysis of genetic association with colorectal cancer (outcome) over genetic association with fasting glucose (exposure).


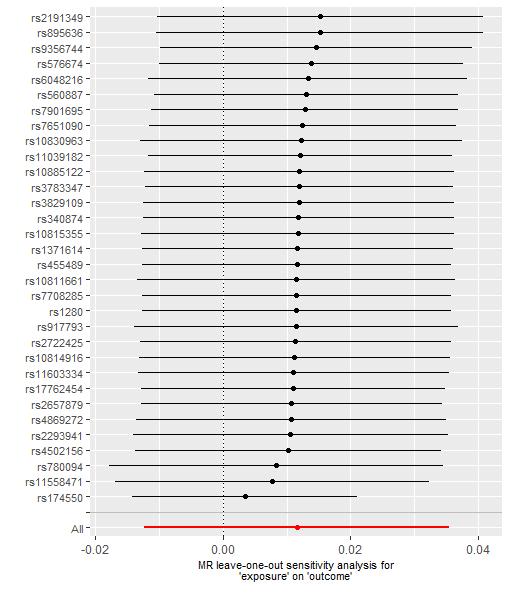


c). Funnel plot of IV precisions (1/SE_IV_) against the IV estimates (β_IV_) for fasting glucose.


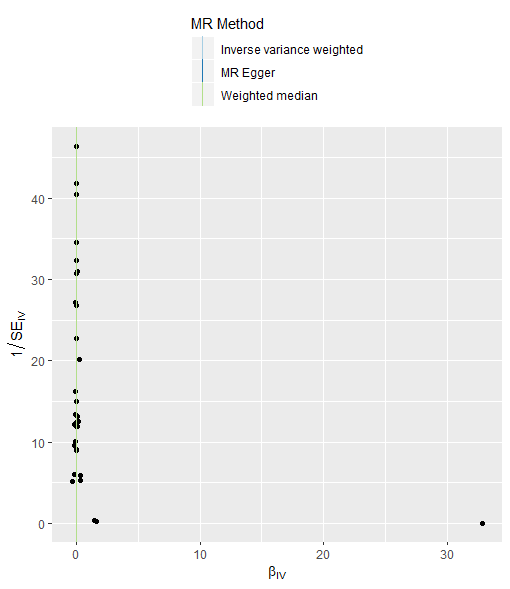


Abbreviation: IV, instrumental variable.

**Supplementary Figure S3. Analyses of HbA1c and colorectal cancer risk**

a). Scatter plot of genetic association with colorectal cancer (outcome) over genetic association with HbA1c (exposure).


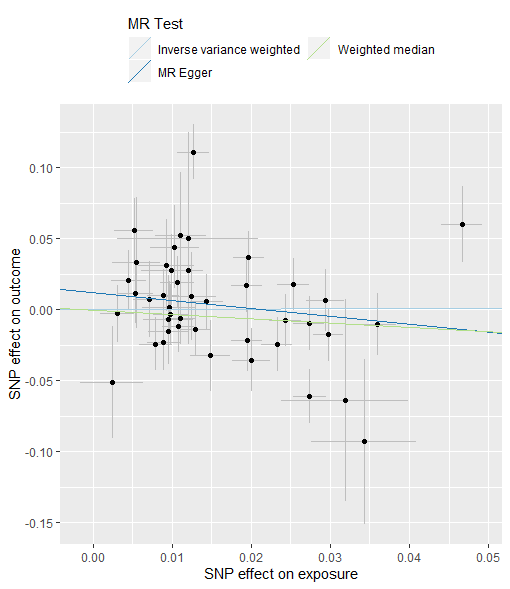


Abbreviation: HbA1c, haemoglobin A1c; SNP, single nucleotide polymorphism.

b). Leave-one-out sensitivity analysis of genetic association with colorectal cancer (outcome) over genetic association with HbA1c (exposure).


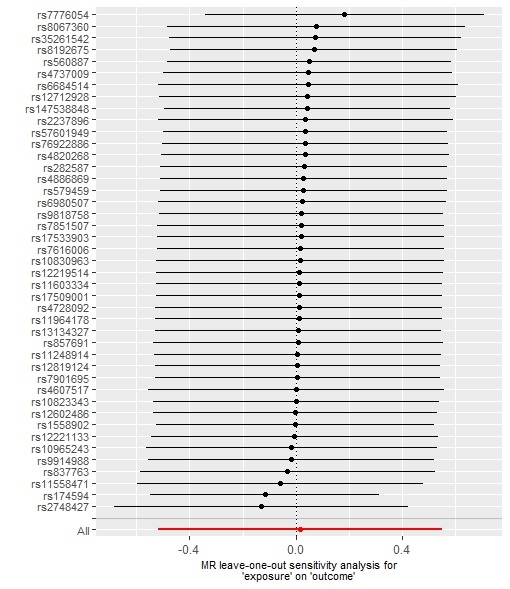


Abbreviation: HbA1c, haemoglobin A1c.

c). Funnel plot of IV precisions (1/SE_IV_) against the IV estimates (β_IV_) for HbA1c.


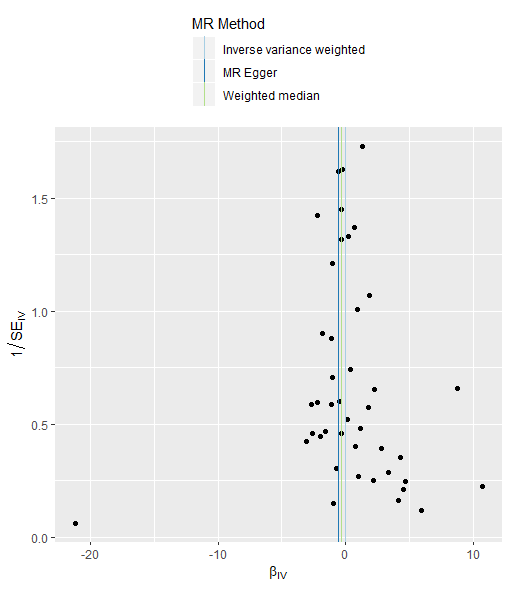


Abbreviation: HbA1c, haemoglobin A1c; IV, instrumental variable.

**Supplementary Figure S4. Analyses of fasting C-peptide and colorectal cancer risk**

a). Scatter plot of genetic association with colorectal cancer (outcome) over genetic association with fasting C-peptide (exposure).


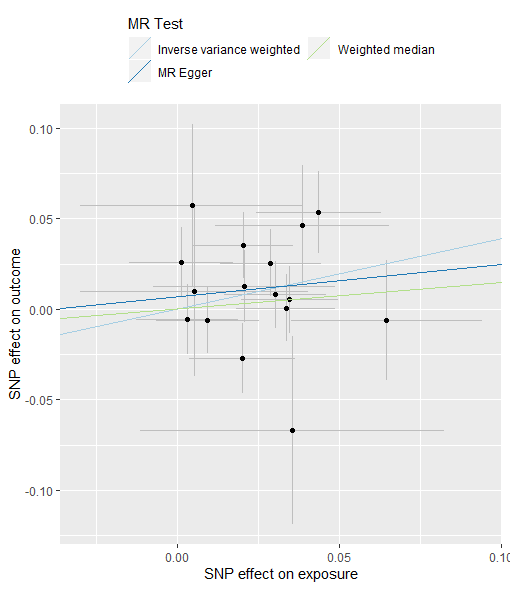


Abbreviation: SNP, single nucleotide polymorphism.

b). Leave-one-out sensitivity analysis of genetic association with colorectal cancer (outcome) over genetic association with fasting C-peptide (exposure).


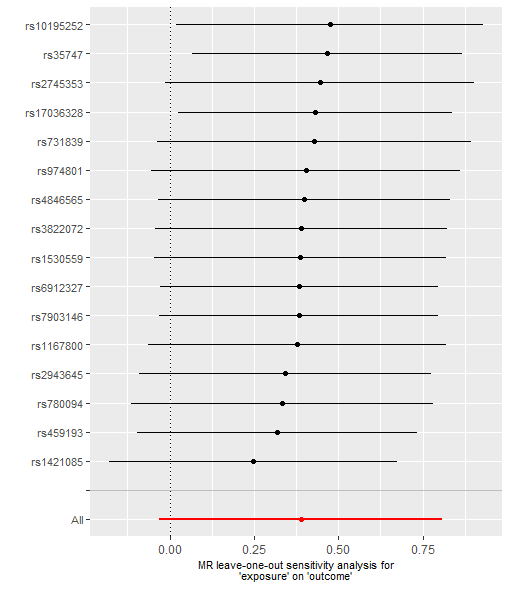


c). Funnel plot of IV precisions (1/SE_IV_) against the IV estimates (β_IV_) for fasting C-peptide.


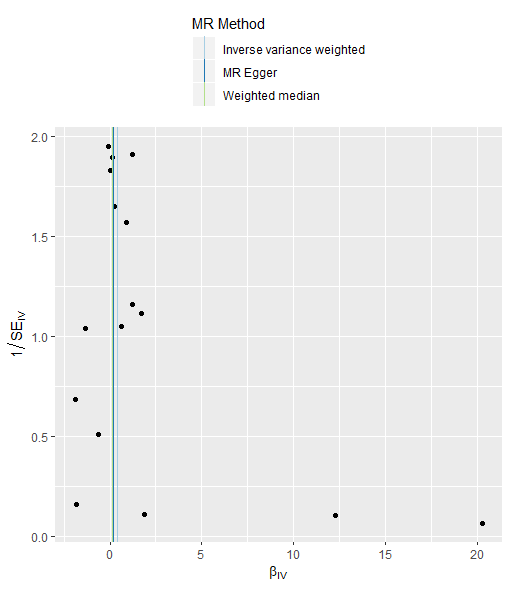


Abbreviation: IV, instrumental variable.
